# PTEN regulates cilia through Dishevelled

**DOI:** 10.1038/ncomms9388

**Published:** 2015-09-24

**Authors:** Iryna Shnitsar, Mikhail Bashkurov, Glenn R. Masson, Abiodun A. Ogunjimi, Sherly Mosessian, Eduardo Aguiar Cabeza, Calley L. Hirsch, Daniel Trcka, Gerald Gish, Jing Jiao, Hong Wu, Rudolf Winklbauer, Roger L. Williams, Laurence Pelletier, Jeffrey L. Wrana, Miriam Barrios-Rodiles

**Affiliations:** 1 Center for Systems Biology, Lunenfeld-Tanenbaum Research Institute, Mount Sinai Hospital, Toronto, Ontario, Canada M5G 1X5; 2 Protein and Nucleic Acid Chemistry Division, Medical Research Council Laboratory of Molecular Biology, Cambridge Biomedical Campus, Cambridge CB2 0QH, UK; 3 Department of Molecular and Medical Pharmacology, University of California, Los Angeles, California 90095, USA; 4 Department of Cell and Systems Biology, University of Toronto, Toronto, Ontario, Canada M5S 3G5; 5 Department of Molecular Genetics, University of Toronto, Toronto, Ontario, Canada M5S 1A8

## Abstract

Cilia are hair-like cellular protrusions important in many aspects of eukaryotic biology. For instance, motile cilia enable fluid movement over epithelial surfaces, while primary (sensory) cilia play roles in cellular signalling. The molecular events underlying cilia dynamics, and particularly their disassembly, are not well understood. Phosphatase and tensin homologue (PTEN) is an extensively studied tumour suppressor, thought to primarily act by antagonizing PI3-kinase signalling. Here we demonstrate that PTEN plays an important role in multicilia formation and cilia disassembly by controlling the phosphorylation of Dishevelled (DVL), another ciliogenesis regulator. DVL is a central component of WNT signalling that plays a role during convergent extension movements, which we show here are also regulated by PTEN. Our studies identify a novel protein substrate for PTEN that couples PTEN to regulation of cilia dynamics and WNT signalling, thus advancing our understanding of potential underlying molecular etiologies of PTEN-related pathologies.

## Abstract (web summary)

The formation of motile cilia is regulated by Dishevelled (DVL), a central component in WNT signalling and planar cell polarity (PCP). Here the authors identify DVL as a novel substrate of the phosphatase PTEN, coupling PTEN to cilia dynamics and PCP.

## Graphical abstract

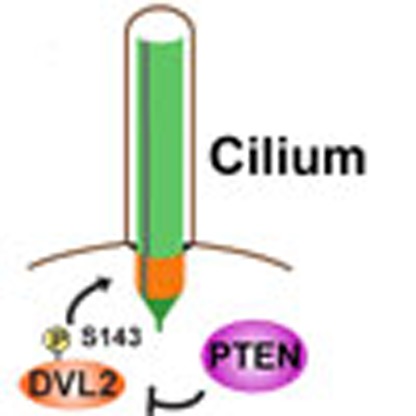

Cilia and eukaryotic flagella are structurally similar organelles that play a pivotal role in eukaryotic biology. Flagella are motile and beat in a propeller-like pattern to drive the movement of single cells, such as spermatozoa. Motile cilia on the other hand, in either mono- or multiciliated cells (MCCs), display coordinated beating movements that are necessary to establish fluid flow across epithelial surfaces, such as respiratory tract or brain ventricles. Another type of cilia are non-motile and play important sensory roles and regulate several signal transduction pathways^1^,^2^. Impairment of cilia function results in a number of human diseases, called ciliopathies, such as Joubert syndrome, Bardet–Biedl syndrome, Meckel–Grüber syndrome, Alström syndrome, polycystic kidney disease and primary ciliary dyskinesia. Defects in cilia formation and function can lead to respiratory abnormalities, hydrocephalus, infertility, deafness, *situs inversus*, formation of kidney and liver cysts, polydactyly, retinopathy and delays in mental development^3^,^4^. Understanding the mechanisms regulating ciliogenesis is thus important in a broad range of human diseases.

Phosphatase and tensin homologue (PTEN) was first described as a dual-specificity phosphatase, able to dephosphorylate lipids and a few protein substrates^5^,^6^,^7^, but the biological importance of PTEN protein phosphatase activity has remained elusive. Indeed, the majority of reports have subsequently focused on PTEN as a lipid phosphatase that dephosphorylates the phospholipid, phosphatidylinositol-3,4,5-trisphosphate (PIP_3_) at position 3 of the inositol ring^8^. By dephosphorylating PIP_3_, PTEN opposes activation of PI3-kinase and inhibits cell growth^9^. Loss of PTEN function is associated with several pathologies including Cowden disease, Bannayan–Riley–Ruvalcaba, Lhermitte–Duclos, Proteus and Proteus-like syndromes, which were recently unified under the name of PTEN hamartoma tumour syndrome (PHTS). PHTS is characterized by tissue overgrowth and increased incidence of tumours in thyroid, breast, prostate, skin and kidney^10^. In addition, patients with PTEN mutations display macrocephaly and sporadic hydrocephalus, congenital defects and delays in mental development, including autism^10^,^11^. This indicates that PTEN has important biological functions in addition to a tumour-suppressive role.

Here we demonstrate that PTEN regulates cilia dynamics and is required for formation of motile multicilia in vertebrates and stabilizes primary cilia in a human retinal epithelial cell line model. Further, we show that during ciliogenesis PTEN controls serine-143 phosphorylation of Dishevelled (DVL)2 that serves as a direct substrate of PTEN *in vitro*. Our studies thus establish a conserved and crucial role for PTEN in cilia dynamics through regulation of DVL phosphorylation.

## Results

### PTEN regulates cilia formation in *Xenopus*

**Figure 1:**
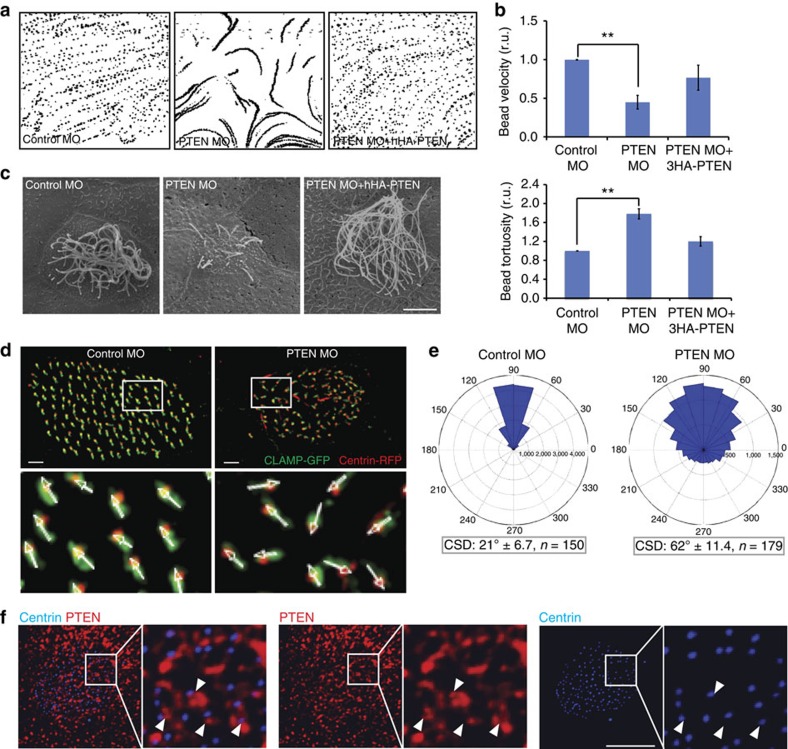
PTEN is required for the formation of multicilia in *Xenopus*. (**a**) Fluorescent beads were applied to the dorsal surface of tadpole embryos, injected with control or PTEN morpholinos (MO) with or without human PTEN mRNA, as indicated. Bead flow was imaged over 10 s (300 frames) and projected bead tracks over 3 s are shown. Note that PTEN knockdown affects speed and directionality of the fluid flow (middle panel). (**b**) Data from **a** were quantified to assess bead velocity and tortuosity (a ratio between total and straight distance of a bead trajectory during a defined time, showing the loss of directionality in movement) relative to controls. Data are plotted as the mean with errors bars representing s.e.m. (*n*=3), **P<0.01 by a *t*-test. (**c**) Cilia defects in tadpole embryo treated as in **a** shown by scanning electron microscopy (scale bar, 5 μm). Images are representative from three independent experiments. (**d**) Control or PTEN morpholino-injected embryos with labelled basal bodies Centrin-RFP and basal rootlets (CLAMP-GFP). Arrows connecting basal bodies (BBs) and basal rootlets (BRs) were manually traced (lower panels). Scale bar, 1 μm. (**e**) Polarization of basal rootlets from embryos in **d** was evaluated. The graphs represent an angle histogram plot of four independent experiments. Before plotting, angles within each cell were subtracted by an average angle of a given cell and normalized to 90°. Angles of BR orientation were also measured, followed by calculation of circular standard deviation (CSD) for each assessed cell. Average CSD±s.d. of CSD across indicated (*n*) number of cells is shown for both control and PTEN knockdown cells. The difference in CSDs between control and PTEN MO-injected cells was analysed using the Mann–Whitney test and found significant (*P*<0.0001). (**f**) 3D reconstruction of the apical surface of a multiciliated cell with white arrows pointing to examples of PTEN (red) localization in close proximity to basal bodies (labelled as Centrin-RFP, blue). Images are representative from three independent experiments. Scale bar, 7 μm.

Previous data indicated that PTEN is important during the early stages of development and axonal guidance in *Xenopus* embryos^12^,^13^. To further elucidate the cellular processes that require PTEN, we injected *Xenopus laevis* embryos with morpholino oligonucleotides, which block the translation of PTEN protein. Embryos, injected in the animal pole with PTEN morpholinos (PTEN morphants) did not display any major developmental defects until late tadpole stages, where approximately 20–25% of embryos showed mild defects in dorsal elongation consistent with potential defects in convergent extension (CE) movements (see below). Furthermore, unlike wild-type (WT) tadpoles, the morphants did not display typical drifting motion. Tadpole drifting is caused by an anterior to posterior fluid flow over the epidermis, so we next examined how PTEN knockdown affected fluid flow over the epidermis by analysing the movement of fluorescent beads on the embryo surface. PTEN loss of function markedly affected bead motility by decreasing velocity and increasing tortuosity (Fig. 1a,b; Supplementary Movies 1–4). Importantly, these effects were rescued by co-injection of a haemagglutinin (HA)-tagged human PTEN construct, which is not targeted by the morpholino (Fig. 1a,b).

Fluid flow over *Xenopus* embryo skin is caused by epidermal MCCs, which bear motile cilia and localize in a ‘salt-and-pepper' pattern along the surface of an embryo^14^,^15^. The structural components of *Xenopus* MCCs are similar to those found in the trachea and brain ventricles of mammals and are critical for creating directional fluid flow^16^. Remarkably, upon analysis of *Xenopus* epidermis by both scanning electron and confocal microscopy, we found that knockdown of PTEN caused defects in the formation of cilia in MCCs (Fig. 1c; Supplementary Fig. 1a). Moreover, cilia formation was rescued by co-injection of HA-tagged human PTEN that is not targeted by the morpholino and possesses 89% identity with its *Xenopus* homologue (Fig. 1c), indicating that the function of PTEN in ciliogenesis is highly conserved across species.

Cilia are dynamic structures that undergo active assembly and disassembly. They emerge from a tubulin-based structure called the basal body (BB) that multiplies in MCCs and subsequently migrates to the apical side. Upon apical docking, BBs create a base for the outgrowth of the axoneme, which protrudes underneath the cellular membrane. BBs also associate with a cytoskeletal structure, called the ciliary or basal rootlet, that extends towards the cell nucleus and is thought to be important for cilia trafficking and stability^17^,^18^. During the establishment of fluid flow, basal rootlets undergo polarization that leads to their unidirectional alignment, thus establishing so-called ‘rotational polarity' that is critical for coordinated movement of cilia^19^. To further address the role of PTEN in cilia formation, we analysed apical docking of BBs and alignment of ciliary rootlets in the morpholino-injected embryos. Analysis of BB positioning both by transmission electron microscopy and confocal microscopy using the BB marker Centrin-red fluorescent protein (RFP) revealed defects in apical docking upon PTEN knockdown (Supplementary Fig. 1b,c). To visualize basal rootlets, embryos were co-injected with the ciliary rootlet marker, CLAMP-green fluorescent protein (GFP), which revealed that basal rootlets in PTEN morphants were not aligned in the same direction at the late tadpole stages when anterior–posterior flow has been established^20^ (Fig. 1d). The misalignment of basal rootlets was significant when quantified as shown in Fig. 1e. Thus, PTEN plays a pivotal role during *Xenopus* ciliogenesis and is important both for apical docking of BBs and subsequently for polarized alignment of ciliary rootlets.

Next, we examined the localization of endogenous PTEN in *Xenopus* epidermis. In MCCs, PTEN was localized ubiquitously, with enrichment on the apical aspect of the cell. PTEN did not co-localize with BB markers, such as gamma-tubulin or RFP-tagged Centrin, but rather was observed adjacent to BBs, as well as in a spotted pattern in the ciliary axoneme itself (Fig. 1f; Supplementary Fig. 1d), suggesting direct functional involvement of PTEN in the regulation of ciliogenesis.

### Loss of *Pten* causes defects in multicilia formation in mice

To study the role of PTEN during cilia formation in mammals, we investigated two types of multiciliated epithelia, tracheal cells in the respiratory tract and ependymal cells lining lateral brain ventricles, both of which are critical for directing fluid flow. In both cell types PTEN shows ubiquitous localization, but upon detailed examination of ependymal cells, similar to *Xenopus* MCCs, Pten was found localized adjacent to BBs (Supplementary Fig. 2b).

**Figure 2:**
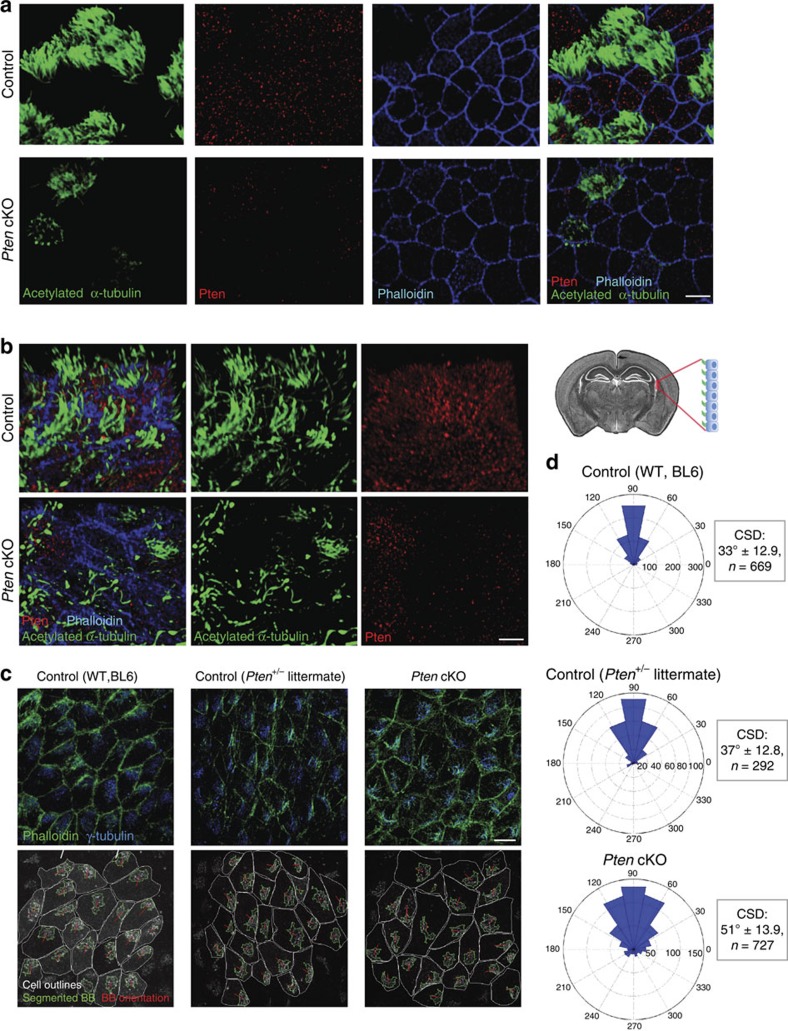
PTEN is required for ciliogenesis in mouse trachea and ependyma. (**a**) Loss of *Pten* causes ciliation defects in trachea. Tracheal multicilia of control littermates (top) or Pten conditional knockout (cKO) embryos (lower panel) were stained with acetylated tubulin (green). To confirm *Pten* loss, cells were stained for Pten (red) and F-actin (Phalloidin, blue). Note that the cell with prominent cilia in *Pten* cKO embryos (upper left) retains residual Pten protein. Images are representative from 15 cKO and 10 control embryos analysed from three independent experiments. Scale bar, 7 μm. (**b**) Loss of *Pten* results in cilia formation defects in ependymal cells (brain localization in schematics on the right). Control or cKO pups, induced with tamoxifen at P0–P2 were analysed for defects in ependymal cilia formation at day 9 of development as in **a**. Images represent 10 cKO and 14 control pups from three independent experiments. Scale bar, 7 μm. (**c**) Basal body (BB) polarization defects in ependymal multiciliated cells (EMCCs) at day 10 of postnatal development. Left panel shows tissue polarization in wild-type mouse of the same genetic background. Middle panel displays BB polarization in EMCC of an animal missing one allele of *Pten*, while the panel on the right displays effect of Pten knockout. Bottom panels illustrate the algorithm for quantification of translational polarity defects, based on Boutin *et al.*^58^ and described in Methods. Scale bar, 10 μm. (**d**) Quantification of *Pten* loss effect on basal bodies translational polarity. The angles for basal body orientation vectors (BBOVs, see Methods) were calculated and angular histogram plots are shown (on average 20 cells per field of view were analysed). Before plotting, angles within each image were normalized to the average angle calculated for each field of view and then underwent 90° rotation. Circular standard deviation (CSD) was calculated for each field of view to assess variation in BBOVs. CSD was estimated for every image plane analysed and compared between genotypes. The difference in BB polarization between control WT and *Pten* cKO was found significant with *P*<0.0001 in an unpaired Mann–Whitney test.

Deletion of *Pten* in mice causes embryonic lethality at early stages of development^21^,^22^. To bypass this, we used a *Pten* conditional knockout allele crossed with tamoxifen-inducible Cre-recombinase under the control of the *FOXJ1* promoter, which is active in cells that develop motile cilia^23^,^24^,^25^. Tamoxifen induction enabled us to analyse the effects of Pten impairment on MCCs from the trachea and ependyma. Tracheal cells initiate expression of FoxJ1 at day 14 of embryonic development and become multiciliated by day 18 (ref. 26). Therefore, to study the effect of Pten loss on the formation of tracheal cilia, mouse dams were injected with tamoxifen on day 14 of pregnancy and embryonic tracheas analysed on day 19, before birth (Supplementary Fig. 2a). Using confocal microscopy on embryonic trachea stained for acetylated tubulin, a cilia axoneme marker, and co-stained for Pten, we observed that loss of Pten expression led to a remarkable decrease in the number and length of multicilia in tracheal cells (Fig. 2a). We next analysed Pten function in ependymal cilia formation. Ependymal cells derive from radial glia cells, which are mono-ciliated and complete the formation of the lateral brain ventricle lining by day 18 of embryonic development^27^. The formation of multiple cilia on ependymal cells occurs in the first 2 days post birth, while proper polarized positioning of BBs and establishment of fluid flow is completed by day 9 of postnatal development^28^. To evaluate the role of Pten during ependyma development, we performed intraperitoneal tamoxifen injections into mouse pups using two different schemes (Supplementary Fig. 2a): (1) inducing *Pten* knockout with tamoxifen at P0–P2 (when multicilia are forming) and analysing the defects at P9, or (2) inducing the knockout at P3–5 (after multicilia are already formed) and analysing the defects at P10. Similar to our observations in tracheal cells, decreased Pten expression drastically affected the number of ependymal cilia (Fig. 2b), which was more apparent during earlier knockout induction.

To establish fluid flow, ependymal MCCs (EMCCs) require two types of polarization. First, BBs migrate towards the anterior side of a cell, creating so-called translational polarity. Next, similar to *Xenopus* MCCs, basal rootlets of cilia within one cell are aligned, creating rotational polarity^29^. Analysis of EMCCs stained with gamma-tubulin, showed that in cells lacking Pten, BB patches localize abnormally and often migrate in a direction different from the neighbouring cell (Fig. 2c; Supplementary Fig. 2b). In addition, a small portion of BB patches retained central localization with just extension towards one side of a cell. This misalignment in BB localization was found significant upon quantification, as shown in Fig. 2d, suggesting that PTEN regulates translational polarity of ependymal cells. Taken together, these data indicate that Pten requirement for ciliogenesis is conserved in mammals.

### PTEN binds DVL

**Figure 3:**
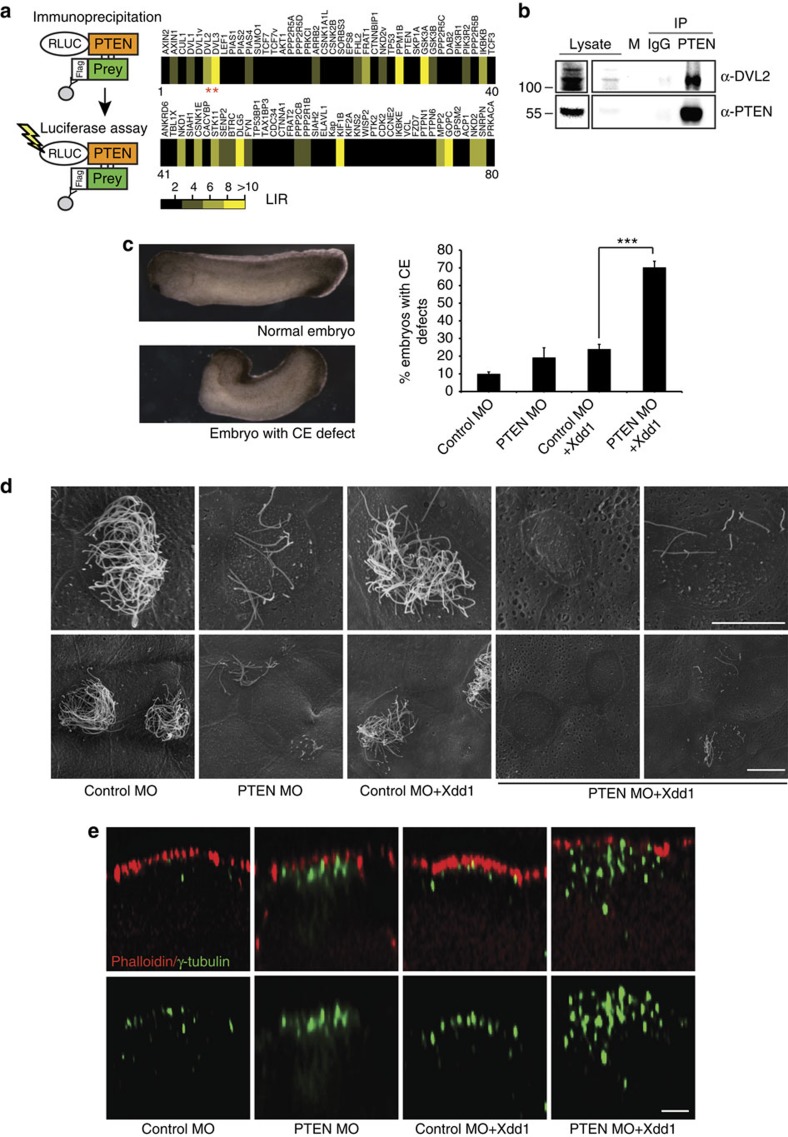
PTEN interacts with Dishevelled-2. (**a**) A LUMIER screen (schematic) was employed to identify novel PTEN-interacting proteins using a collection of eighty 3Flag-tagged preys as indicated. Results of an average of two independent LUMIER screens are plotted as a heatmap of the Luminesence Intensity Ratio (LIR, color scale). Both DVL2 and DVL3 (red stars) were identified as PTEN interactors. DVL1v and TCF7v denote splice variants. (**b**) Interaction between endogenous PTEN and DVL2. Cell lysates were immunoprecipitated with PTEN or control (IgG) antibodies and blotted for DVL2 (top panel) or PTEN (bottom panel), as indicated. An aliquot of total cell lysate and molecular weight markers (M) were loaded on the left side of the gel. The separate panels to the left show darker exposures of total cell lysate samples from the same blots. Image is representative of five independent experiments. (**c**) PTEN functions in convergent extension (CE) morphogenetic movements. *Xenopus* embryos injected with control or PTEN morpholinos either alone or together with low doses (100 pg) of dominant-negative Xdd1 Dishevelled, were then scored for CE defects (dorsal elongation and neural tube closure defects) as illustrated on the left. The graph displays the mean with error bars representing s.e.m. for Xdd1 (*n*=4) with 100 and more embryos counted for every condition (***P<0.001 by a *t*-test). Note that interference with PTEN strongly synergizes with Xdd1 to induce CE defects. (**d**) Scanning electron micrographs of multiciliated cells in the epidermis of *Xenopus* embryos treated as in **c**. Note the enhanced cilia defects in the embryos co-injected with PTEN MO and low doses of Xdd1 (scale bar, 10 μm). Images are representative from three independent experiments. (**e**) Basal body (BB) docking defects in embryos co-injected with PTEN MO and Xdd1. BBs in the ectoderm of *Xenopus* embryos treated as in **c** were stained for gamma-tubulin (green) and counterstained for cortical F-actin to visualize the apical surface of the cell (Phalloidin, red). Apical–basal reconstruction of confocal images reveals enhanced BB docking defects when Xdd1 was co-injected with PTEN MO (scale bar, 4 μm). Images are representative from at least three biological replicates.

To uncover how PTEN might regulate cilia formation and function, we searched for novel PTEN-binding partners using an automated luminescence-based mammalian interactome (LUMIER) assay^30^. PTEN fused to Renilla luciferase was used as a bait against a collection of 3Flag-tagged preys^31^ (Fig. 3a). This confirmed interactions with known partners of PTEN, such as LKB1 (STK11), and PIK3R2, as well as casein kinase 1e (CSNK1E) (http://thebiogrid.org). We also identified novel interactions with the DVL proteins (Fig. 3a), of which DVL2 and DVL3 displayed the strongest association with PTEN.

DVL is a major component of Wnt signalling pathways, and contains several functional domains, such as the DIX domain, a protein-binding PDZ domain, a DEP domain and an unstructured C terminus^32^. We verified the DVL2–PTEN interaction by manual LUMIER analysis and performed co-immunoprecipitation (IP) experiments using differentially tagged proteins (Supplementary Fig. 3a,b). Domain mapping of DVL2 mutants^31^ further showed that the C-terminal part of DVL2 including the DEP domain was required for PTEN interaction (Supplementary Fig. 3c,d). Endogenous PTEN and DVL2 also interacted in a human retinal epithelial cell line (hTERT-RPE1) that assembles cilia *in vitro* (Fig. 3b). Moreover, endogenous PTEN partially co-localized with ectopically expressed DVL in regions adjacent to BBs in hTERT-RPE1 (data not shown) and with *Xenopus* Dishevelled (Dsh) in MCCs (Supplementary Fig. 2c). To better visualize co-localization between PTEN and Dsh in *Xenopus* MCCs, we also used a DVL mutant lacking the DIX domain (dDIX-Dsh). This mutant retains proper localization to the BB area and does not form aggregates, which are frequently detected when WT Dsh is overexpressed^20^. Interestingly, in *Xenopus* MCCs PTEN and dDIX-Dsh-GFP were localized next to BBs marked by Centrin-RFP and were typically observed in adjacent regions that partially overlapped. Thus, overlapping PTEN and Dsh signals (Supplementary Fig. 2c, white arrows), as well as adjacent localization (magenta arrows) were readily apparent.

### PTEN–Dsh interaction regulates morphogenetic processes

DVL is a particularly intriguing PTEN partner, as it is crucial for cilia formation in both *Xenopus* and mice. In particular, similar to the effects of *PTEN* knockdown described above, loss of DVL affects apical docking of BBs and overexpression of a dominant-negative DVL induces rotational polarity defects^20^. To explore the physiological relevance of PTEN–DVL interaction, we co-injected PTEN morpholino together with either full-length GFP-Dsh or lower doses of a dominant-negative version Xdd1 (ref. 33) and analysed the morphological changes in *Xenopus* embryos, specifically focusing on defects in ciliogenesis and CE movements. CE movements are regulated by DVL and comprise a polarized motility within designated groups of cells that leads to their ordered intercalation with each other, resulting in tissue elongation during gastrulation and neural tube closure^34^. Overexpression of either lower doses of a dominant-negative DVL (Xdd1)^33^, or full-length GFP-tagged DVL combined with PTEN loss of function, markedly increased the number of embryos bearing CE defects (Fig. 3c; Supplementary Fig. 3e). Given the importance of DVL dosage and localization during CE movements, similar effects from Xdd1 and Dsh-GFP can be expected. Moreover, when the embryos co-injected with Xdd1 and PTEN morpholino were analysed for cilia formation, we observed enhanced cilia loss, as well as more severe BB docking defects, in comparison with either treatment alone (Fig. 3d,e). These data suggest that DVL interaction with PTEN is important for its planar cell polarity (PCP) functions in CE movements and for BB docking during cilia formation.

### PTEN regulates cilia dynamics via DVL2 phosphorylation

**Figure 4:**
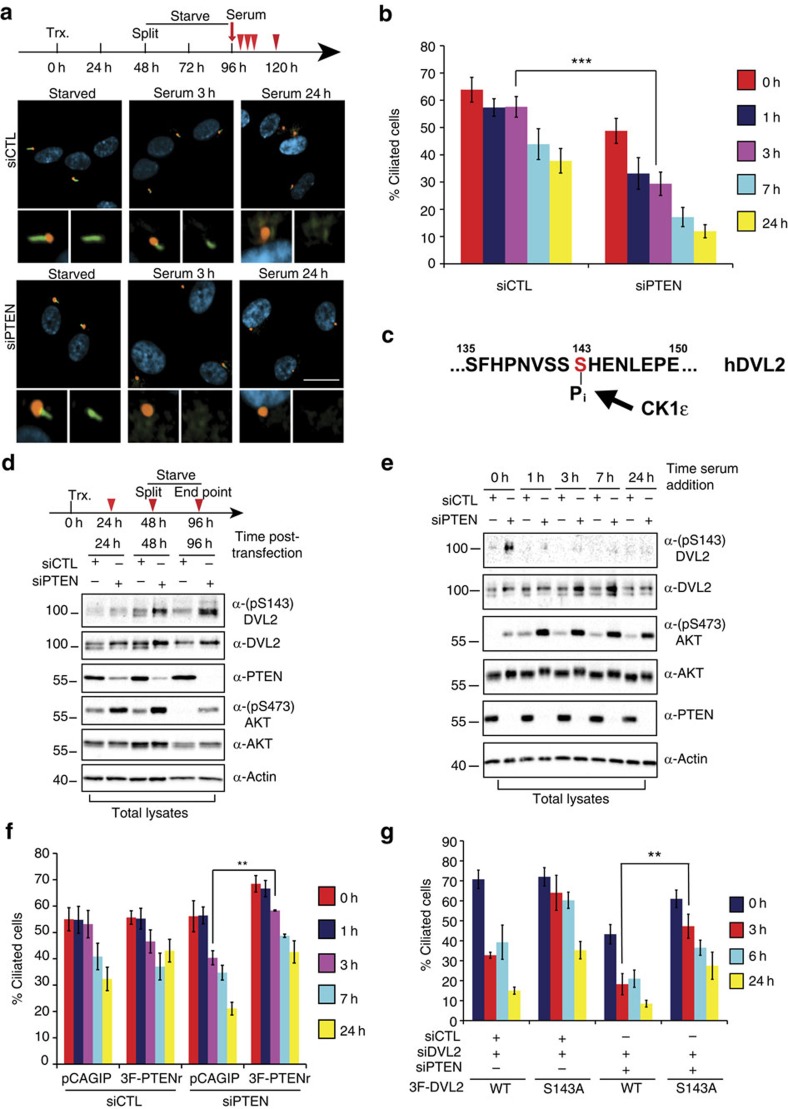
PTEN regulates cilia disassembly and phosphorylation of DVL2. (**a**) Top: time-course scheme for cilia disassembly in hTERT-RPE1 cells transfected (Trx.) with siControl (siCTL) or siPTEN. Bottom: representative images of cells starved and after serum addition, stained for acetylated tubulin (cilia axoneme, green), pericentrin (centrioles, red), 4′,6-diamidino-2-phenylindole (DAPI, cell nuclei, blue); scale bar, 20 μm. (**b**) PTEN loss promotes cilia disassembly. The percentage of ciliated cells from **a** was quantified by automated imaging (Supplementary Fig. 4 and Methods). Graph shows mean with error bars (s.e.m.) from *n*=7 (*n*=4 for a 7-h time point), with ∼1,000 cells for each condition (***P<0.001 by a *t*-test). (**c**) DVL2 peptide sequence containing the serine-143 phosphorylation site (in red), recognized by the antibody. (**d**) PTEN knockdown enhances DVL2 phosphorylation on serine 143 during cilia formation. hTERT-RPE1 cells were transfected with siCTL or siPTEN and lysates were analysed at 24, 48 and 96 h post transfection. Cells were starved for the last 48 h before lysis. (**e**) Phospho-S143-DVL2 levels during cilia disassembly. The experiment was performed as in **a** with siCTL- or siPTEN-transfected hTERT-RPE1 cells. Cell lysates were analysed by immunoblotting using the indicated antibodies. Samples in **d** and **e** are representative from at least four independent experiments. (**f**) siRNA-resistant PTEN rescues accelerated cilia disassembly caused by PTEN knockdown. Cilia disassembly was evaluated in hTERT-RPE1 lines expressing siRNA-resistant Flag-tagged PTEN (3F-PTENr) or empty vector (pCAGIP), upon transfection with siCTL or siPTEN. Cilia disassembly was quantified as in **b**. Results are plotted as mean with error bars showing s.e.m.; *n*=4, **P<0.01 by a *t*-test. (**g**) Expression of DVL2 S143A rescues the effect of PTEN knockdown on cilia disassembly rates. Cilia disassembly was performed in hTERT-RPE1 stable cell lines expressing either 3F-DVL2 or its S143A mutant in the presence of siDVL2 (targeting its endogenous 3′-untranslated region) upon siPTEN or siCTL transfection. Cilia disassembly was quantified as in **b** and is plotted as percentage of ciliated cells (mean with error bars showing s.e.m. of *n*=4; **P=0.01 by a *t*-test) with an average of 170 cells analysed per condition in each experiment.

In addition to its role during ciliogenesis in *Xenopus*, DVL also regulates cilia dynamics in human retinal pigmented epithelial cells (hTERT-RPE1)^35^. Therefore, we next used these cells to further explore the role of the PTEN–DVL2 axis. When cultured in the absence of serum, which inhibits cell division, the majority of hTERT-RPE1 cells form a single cilium within 24–48 h. Re-addition of serum results in rapid disassembly of cilia as cells re-enter the cell cycle^36^. To quantify cilia dynamics in hTERT-RPE1 cells, we developed an automated image analysis algorithm employing acetylated α-tubulin staining to mark axonemes and counterstained with pericentrin to mark the ciliary BB (Supplementary Fig. 4a). Although the percentage of ciliated cells was not significantly altered in PTEN short interfering RNA (siRNA)-transfected cells after 48 h of starvation, upon serum addition, cells lacking PTEN disassembled cilia at an increased rate, compared with control cells (Fig. 4a,b; Supplementary Fig. 4b). Consistent with an increased cilia disassembly rate, we also observed a more rapid decrease in cilia length in cells lacking PTEN (Supplementary Fig. 4c). Furthermore, in the absence of PTEN, peak disassembly occurred early, within the first 2–3 h after serum addition, while in control cells, cilia loss started later, being noticeable at 7 h post serum treatment (Fig. 4a,b). After 7 h, the dynamics of cilia loss was comparable in control cells and those lacking PTEN (Fig. 4b; Supplementary Fig. 4b). Of note, the effect of PTEN knockdown was rescued by stably expressing siRNA-resistant 3Flag-PTEN (Fig. 4f; Supplementary Fig. 5a–e). Importantly, PTEN knockdown did not accelerate cell cycle in hTERT-RPE1 cells nor increased mitotic cell numbers in P9–P10 mouse ependymal cells (Supplementary Fig. 6a,b). Collectively, these data demonstrate that PTEN inhibits cilia disassembly, independent from regulating cell cycle entry.

Previous reports indicate that cilia disassembly is triggered by casein kinase 1 (CK1ɛ)-dependent DVL2 phosphorylation on serine 143 (Fig. 4c) that promotes DVL2 interaction with Polo-like kinase-1 (PLK1), which subsequently activates the HEF-1/Aurora kinase A/HDAC6 pathway, triggering tubulin deacetylation and cilia disassembly^35^,^36^. These observations prompted us to test whether PTEN can regulate phospho-DVL2 levels. First we compared the levels of phosphorylated serine 143 (pS143) on DVL2 in hTERT-RPE1 cells during the formation of cilia in control cells versus cells lacking PTEN. This revealed that PTEN knockdown strongly increased levels of pS143-DVL2 (Fig. 4d) that was rescued by re-expressing a siRNA-resistant PTEN variant (Supplementary Fig. 5d,e). Strikingly, after serum addition, levels of phosphorylated serine 143 markedly decreased within the first hour (Fig. 4e), suggesting that DVL2 phosphorylation is important primarily during the initiation of disassembly and consistent with PTEN loss accelerating disassembly during the early phase (Supplementary Fig. 4b). Next, we overexpressed a DVL2 S143A mutant, which cannot be phosphorylated at serine 143, in hTERT-RPE1 cells lacking both endogenous PTEN and DVL2. This revealed efficient rescue of the increased cilia disassembly rate caused by PTEN knockdown when compared with cells expressing WT DVL2 (Fig. 4g; Supplementary Fig. 7a,b). Consistent with our *in vitro* findings, overexpressed DVL2 S143A, but not DVL2 WT, reversed the effects of PTEN morpholino on multicilia assembly in *Xenopus* (Supplementary Fig. 7c,d). These results indicate that PTEN regulates cilia disassembly via S143 phosphorylation of DVL2.

**Figure 5:**
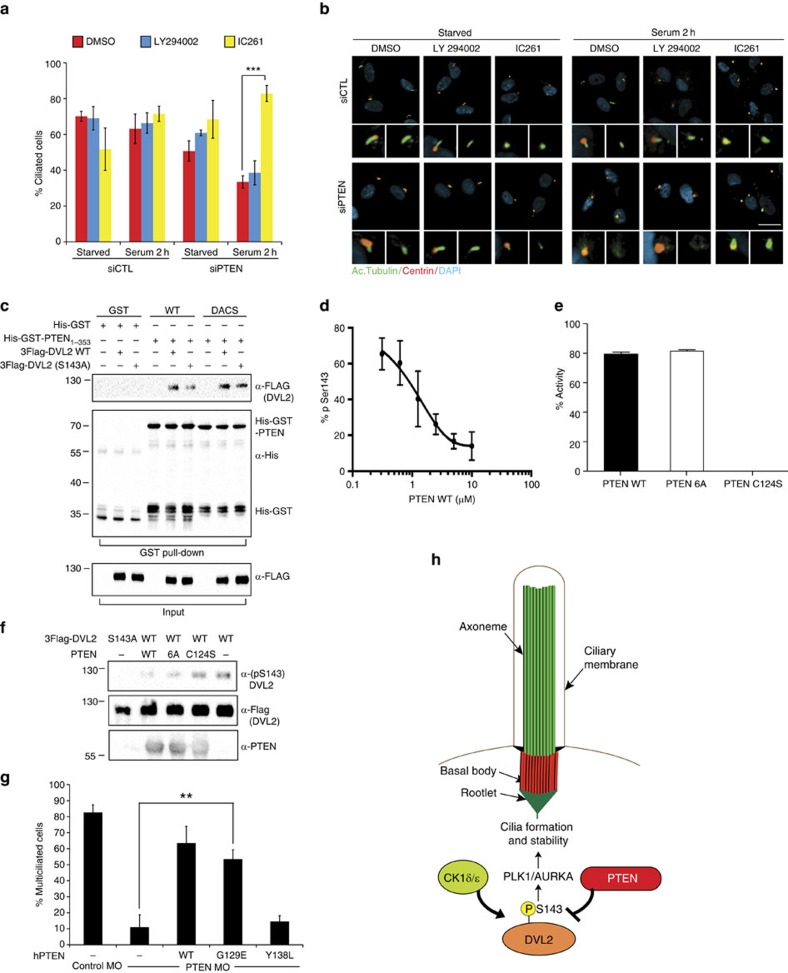
PTEN affects ciliogenesis by targeting DVL2. (**a**) PTEN-dependent cilia disassembly is blocked by CK1δ-ɛ inhibitor but not by PI3-kinase inhibitor. hTERT-RPE1 cells transfected as in Fig. 4a were treated with 10 μM of LY294002 (PI3K inhibitor) or 40 μM of IC261 (CK1δ-ɛ inhibitor) for a total of 4 h (2 h before plus 2 h post serum addition). Ciliation was quantified at 0 h (starved) and 2 h of disassembly. Graph represents three independent experiments; data are mean with error bars showing s.e.m.; ***P<0.001 by a *t*-test, with 300 cells or more per condition. (**b**) Representative images from **a** stained for acetylated tubulin (axonemes, green) and pericentrin (centrioles, red). Scale bar, 20 μm. (**c**) DVL2 binds PTEN *in vitro*. hTERT-RPE1 cells overexpressing 3Flag-DVL2 wild-type (WT) or S143A mutant were lysed and precipitated using glutathione-sepharose beads coupled to either His-GST-PTEN_(1–353)_ WT or DACS mutant and analysed by immunoblotting. (**d**,**e**) PTEN dephosphorylates DVL2 pSerine-143 peptide. (**d**) Dephosphorylation of pS143-peptide (Fig. 4c) was analysed in an enzyme-linked immunosorbent-type assay, after 10 min incubation with varied concentrations of PTEN WT, purified using baculoviral system. The percentage of remaining phospho-serine-143 was quantified by normalizing to a condition containing the peptide without PTEN. Experiments were performed in triplicates. Results are plotted as the mean with error bars indicating s.d. (*n*=3). (**e**) pS143 dephosphorylation efficiency by PTEN variants (10 μM) was compared with PTEN WT as in **d**. Experiments were carried out in triplicate, *n*=3. (**f**) PTEN dephosphorylates DVL2 at serine 143. Immunoprecipitated 3Flag-DVL2 from HEK293T cells was incubated with PTEN variants for 1 h. Serine-143 phosphorylation was analysed by immunoblotting. Figure represents three independent experiments. (**g**) A protein phosphatase dead PTEN mutant (Y138L) fails to rescue multicilia defects in *Xenopus*. Effects on multicilia were quantified by visual analysis of acetylated tubulin (axoneme) staining of embryos' epidermis. Embryos were injected with indicated morpholinos, hPTEN constructs and Centrin-RFP as a lineage tracer. The percentage of normal multiciliated cells was plotted as mean with error bars in s.e.m. from four independent experiments; **P<0.01 by a *t*-test, with more than 500 cells per condition. (**h**) Model for PTEN function during cilia formation and stability.

PTEN is primarily described as a lipid phosphatase, with many intracellular functions mediated through the dephosphorylation of PIP_3_ and consequent inhibition of the PI3-kinase signalling pathway^37^. To define whether PTEN stabilization of cilia might also involve suppression of PI3K signalling, we attempted to rescue the PTEN knockdown cilia phenotype using the PI3-kinase inhibitor LY294002 (ref. 38). Remarkably, accelerated cilia disassembly was not rescued by LY294002 treatment, despite profound inhibition of the PI3K downstream target, activated Akt (Fig. 5a,b; Supplementary Fig. 8a,b). Similarly, the loss of cilia in *Xenopus* MCCs upon PTEN knockdown was not rescued by LY294002 treatment (Supplementary Fig. 8c). These results indicate that increased cilia disassembly rate caused by PTEN knockdown is independent from its role in regulating PIP_3_ levels and PI3K signalling.

CK1ɛ phosphorylates Ser143 on DVL2 (ref. 35), thus we next tested the effect of IC261, an inhibitor of CK1ɛ^39^ on PTEN-mediated cilia disassembly. Remarkably, treatment of cells with IC261 decreased DVL2 pS143 levels and concomitantly reversed the accelerated cilia disassembly caused by PTEN knockdown (Fig. 5a,b). Interestingly, IC261 also inhibits the CK1δ isoform, which promotes cilia length^35^, and consistent with this, we observed cilia shortening in IC261-treated cells that was independent of the PTEN knockdown (Supplementary Fig. 8a). Phosphorylation of DVL2 was shown to induce cilia disassembly by stimulating a PLK1/AURKA cilia disassembly pathway^35^. Therefore, we tested inhibition of this downstream pathway. This revealed that inhibition of both kinases efficiently reversed accelerated disassembly caused by PTEN knockdown, while inhibitors to either kinase alone led to partial reversal (Supplementary Fig. 9a,b). These results indicate that PTEN-dependent stabilization of cilia is mediated via regulation of CK1ɛ-dependent phosphorylation of DVL and the PLK1/AURKA disassembly pathway.

### PTEN dephosphorylates serine 143 of DVL

Since the effect of PTEN knockdown on S143 phosphorylation of DVL2 and increased cilia disassembly appeared independent from PI3K signalling, we aimed to determine whether PTEN might directly dephosphorylate DVL2. First, we examined whether recombinant PTEN binds 3Flag-DVL2 and its S143A mutant *in vitro*. For this purpose, both DVL2 constructs were overexpressed in hTERT-RPE1 cells and were then precipitated using bacterially purified WT GST-PTEN, or a catalytically inactive D92A/C124S (DACS) mutant. Both DVL2 WT and S143A mutant bound WT PTEN and the DACS mutant with comparable efficiencies (Fig. 5c). Next, we tested the activity of WT PTEN and previously characterized mutants that lack either protein phosphatase (Y138L)^40^,^41^, lipid phosphatase (G129E)^42^ or both activities (C124S) towards phosphorylated serine 143 on DVL2. For this purpose, we developed an *in vitr*o enzyme-linked immunosorbent assay-based test employing purified PTEN from a baculoviral expression system and an 18-amino-acid peptide containing the pS143 on DVL2 (Fig. 4c). This revealed that PTEN efficiently dephosphorylated the synthetic peptide, with a half-maximal activity of ∼1 μM PTEN (Fig. 5d). Notably, both PTEN WT and the constitutively active PTEN 6A mutant^41^,^43^ efficiently dephosphorylated the pS143 peptide, as did the G129E lipid phosphatase mutant albeit at 87% of WT PTEN activity. Crucially, no dephosphorylation was observed in the presence of the catalytically inactive PTEN C124S nor the protein phosphatase dead Y138L mutants (Fig. 5e; Supplementary Fig. 10a). We also analysed dephosphorylation of the pS143 peptide by mass spectrometry and observed a dephosphorylated product only upon incubation of the peptide with PTEN, with the dephosphorylation reaction efficiently blocked by the PTEN-specific inhibitor, VOOH-pic (Supplementary Fig. 10b)^44^. Finally, we incubated 3Flag-DVL2 immunoprecipitated from HEK293T cells, with purified PTEN. Similar to our analyses of the synthetic peptide, PTEN WT or the activated 6A mutant, but not the inactive C124S mutant, catalysed dephosphorylation of full-length DVL2 (Fig. 5f). Next, we tested whether these mutants could rescue cilia defects in *Xenopus* PTEN morphants. Importantly, these data show that the lipid phosphatase-inactive mutant (G129E), but not the protein phosphatase-inactive mutant (Y138L), rescued multicilia defects (Fig. 5g; Supplementary Fig. 10c). We also analysed regulation of DVL2 serine-143 phosphorylation by overexpressing PTEN or its mutant derivatives using lentiviral transduction of cells with or without PTEN knockdown. This showed that while both PTEN WT and the lipid phosphatase-inactive G129E mutant suppressed phosphorylation of DVL2 on serine 143 that accumulated upon PTEN knockdown, the C124S and Y138L mutants did not (Supplementary Fig. 10d). Taken together, these results demonstrate that PTEN regulates DVL2 serine-143 phosphorylation, which is a critical site for DVL2-mediated cilia destabilization and disassembly.

## Discussion

In the present work, we demonstrate an unexpected function for PTEN in the regulation of cilia formation in *Xenopus* epithelium as well as mouse trachea and ependyma. Cilia are dynamic structures whose formation and steady-state length depend on balanced assembly and disassembly rates that rely on intraflagellar transport machinery^45^. As cilia start to form, the assembly rate dominates and is facilitated by anterograde intraflagellar transport machinery, which decreases when cilia reach a steady-state length. Conversely, if disassembly rates increase, cilia will shorten and eventually disappear^46^. Our results using an *in vitro* epithelial cell model identified DVL2 as a novel PTEN-binding partner and protein substrate. In particular, we demonstrated that PTEN suppresses CK1ɛ-dependent phosphorylation of DVL2, which otherwise promotes cilia disassembly via PLK1/AURKA signalling. We therefore propose that PTEN promotes cilia formation by stabilizing cilia against CK1ɛ-DVL2-mediated disassembly (Fig. 5h). Finally, it is important to point out that while our studies show that PTEN can directly dephosphorylate DVL2 *in vitro*, it is possible that regulation of serine-143 phosphorylation of DVL2 by PTEN in cells may require additional protein factors, or post-translational modifications. Furthermore, PTEN may also regulate other pathways in addition to DVL that are important in cilia formation and stability.

During the process of cilia formation, other structures at the cilia base, such as basal rootlets, are crucial for the establishment of proper protein trafficking into the cilia and their ablation or mis-positioning, can also result in enhanced cilia loss^17^,^18^. We showed that in *Xenopus* embryos, PTEN loss causes defects in BB positioning and alignment of basal rootlets. Therefore, the enhanced cilia disassembly noted in hTERT-RPE1 cells in the absence of PTEN might reflect improper protein trafficking into cilia, which is also consistent with the observed rapid decrease in cilia length. Indeed, a link between ciliogenesis and the trafficking machinery has been previously demonstrated in *Xenopus*, as DVL morphants display a marked increase of apical cytoplasmic vesicles associated with ciliary loss^20^. DVL2 phosphorylation on Ser143 by CK1ɛ is also critical for the regulation of cilia, as it promotes binding to PLK-1 and subsequent activation of aurora kinase A, which drives cilia disassembly^35^. Our data indicate that while pS143-DVL2 accumulates during cilia formation in starvation conditions, PTEN nevertheless suppresses the steady-state level of phosphorylation. Thus, phosphorylation of DVL balanced by CK1ɛ and PTEN modulates PLK signalling, providing dynamic regulation of cilia formation in diverse conditions. In this model, PTEN stabilizes unphosphorylated DVL2 to favour axoneme formation as well as proper BB and basal rootlet positioning (Fig. 5h). Furthermore, although phospholipid production by INPP5e or PI3K C2α has been previously linked to cilia formation and stability^47^,^48^, our data indicate that the lipid phosphatase activity of PTEN is not involved in cilia stabilization. It is likely that the type of phospholipid messengers, along with the specific temporal and spatial localization of the enzymes, define the roles of INPP5e and PI3K C2α on cilia dynamics.

Our findings provide important contributions to our understanding of PTEN roles in PHTS-related pathologies. In particular, conditional ablation of PTEN in mice causes hydrocephalus^49^, which can result from defects in cerebrospinal fluid flow due to cilia instability in the ependyma lining of the brain ventricles^50^. In addition, recent studies have linked problems in ciliogenesis to autism^51^, which is also associated with PTEN mutations in children^11^,^52^. Our results now imply a link between PTEN function and the genetically complex ciliopathies, and suggest the importance of examining PTEN for mutations in patients exhibiting such disorders^53^. Further research on PTEN-dependent molecular mechanisms underlying these pathologies may thus lead to novel therapies. Our work also identifies DVL2 as a novel PTEN target^6^,^7^, and we demonstrated that the loss of PTEN function resulted in rotational polarity defects and abnormal BB positioning in multiciliated epithelia. These phenotypes were previously reported for other components of the PCP pathway and thus linked PCP to ciliogenesis^54^. As loss of PTEN strongly enhanced CE defects in *Xenopus* caused by expression of a dominant-negative DVL mutant, our studies now define a role for PTEN–DVL in the regulation of polarized cell movements. Thus, in addition to its role in cilia dynamics, we propose PTEN as a novel regulator of vertebrate PCP signalling. Moreover, as stable cilia are lost in many tumour cells, and PCP has recently emerged as a key pathway controlling metastatic phenotypes^55^, our study points to additional tumour suppressor functions for PTEN that lie beyond inhibition of the PI3K pathway.

## Methods

### *Xenopus* embryos micromanipulations

To prepare RNA for microinjections, synthetic capped messenger RNAs (mRNAs) were made using the SP6 mMESSAGE mMACHINE Kit (Life Technologies, cat# AM1340) according to the manufacturer's instructions from corresponding templates linearized with NotI (NEB), except the PTEN constructs, which were linearized using Asp718I (Roche). To knockdown PTEN function in *Xenopus* embryos, we used previously reported PTEN morpholino (PTEN-MO) oligonucleotides^13^ 5′-CGAACTCCTTGATGATGGCGGTCAT-3′. As a control, we used mismatched PTEN morpholino (control MO) with five substituted oligonucleotides, indicated in small letters: 5′-CGAACTCgTTcATcATGcCcGTCAT-3′. Morpholinos were synthesized by Gene Tools and handled according to the manufacturer's instructions. To obtain eggs for microinjections, *X. laevis* females were injected with 600 U of human chorionic gonadotropin (Sigma-Aldrich, SG-10) 12–16 h before an experiment. Spawned eggs were fertilized *in vitro* in the 0.1 × MBSH buffer containing 10 mM Hepes (pH 7.4), 88 mM NaCl, 1 mM KCl, 2.4 mM NaHCO_3_, 0.2 mM MgSO_4_, 0.41 mM CaCl_2_ and 0.66 mM KNO_3_. After removal of jelly coats with 2% cystein chloride solution, pH 8.0, embryos were rinsed four times with 0.1 × MBSH buffer and transferred into 1 × MBSH buffer with 4% Ficoll PM 400 (Sigma-Aldrich, cat# F4375) for microinjections. Embryos were injected at the animal pole with 5 nl of mRNA or morpholino solution in each cell at the two-cell stage. The following amounts of mRNAs and morpholinos were used for microinjections: Dsh-GFP 100 pg, Xdd1 100 pg, human Flag-tagged DVL2 and its S143A mutant 100 pg, CLAMP-GFP 100 pg, Centrin-RFP 100 pg, PTEN-MO 40 ng, control-MO 40 ng, human PTEN HA-tagged, as well as its G129E and Y138L mutant versions, 250 pg, per embryo. For CE assessment, more than 30 embryos per condition were analysed; for the analysis of ciliation defects, on average 15 image planes taken from 4–5 different embryos per condition were analysed. To assess fluid flow, we immobilized embryos in holes made in 0.7% agarose and added Fluorescent Carboxylate-Modified Microspheres (Life Technologies, F-8813). The fluid flow was analysed by movement of Microspheres at the dorsal side of an embryo, which was recorded on Hamamatsu EMCCD Digital camera (C9100-13) connected to either a Axiovert 200 M, Zeiss or a DMIRE2, Leica microscope with a spinning disk confocal scanner (CSU10, Yokogawa). The imaging was done at 37 frames per second and acquired using Volocity Software (PerkinElmer). To calculate velocity and tortuosity of bead movement, movie frames were exported as a series of tiff files and analysed by ImageJ (http://imagej.nih.gov/ij/). For all experimentation involving microscopy analysis, at least five *Xenopus* embryos per condition were used. All sample sizes are specified in each figure legend. No specific blinding/randomization strategies were used, but all samples were equally treated and imaged under similar conditions.

### Plasmids for *Xenopus* work

All constructs used for microinjections in *Xenopus* embryos had pCS2+ backbone. *Xenopus* DVL constructs and Centrin-RFP were obtained from Addgene: Xdsh-GFP (Addgene Plasmid 16788), Xdd1 (Addgene Plasmid 15491), Centrin-RFP (Addgene Plasmid 26753). CLAMP-GFP was generously provided by the Dr J. Wallingford group^20^. HA-tagged and Flag-tagged versions of PTEN were subcloned into PCS2+ vector as follows: PTEN-coding sequence with a tag was excised from pCMV5B vector using KpnI and HindIII restriction sites, blunted with Klenow fragment (Fermentas) and inserted into blunted pCS2+ vector, digested with BamHI. For overexpression in *Xenopus*, the Flag-tagged versions of human DVL2 protein were subcloned into pCS2+ vector from the original pCMV5 constructs, using KpnI/Asp718I (Roche) and XbaI restriction sites. DVL2 fragments were subsequently blunted using Klenow fragment (Fermentas) and inserted into pCS2+ vector backbone, linearized by BamHI.

### Mouse work

All procedures were done in compliance with standardized animal use protocols at the Toronto Centre for Phenogenomics. FOXJ1-CreER mouse line^24^ was generously provided by Dr Brigit L. Hogan. FOXJ1-CreER mice were crossed with a previously published Pten^tm1Hwu^ line^23^, obtained through The Jackson laboratory (Stock number 006440), which enables a conditional knockout of the *Pten* gene. The first generation of males, which were positive for FOXJ1-Cre and bearing one Pten^tm1Hwu^ allele were crossed with Pten^tm1Hwu^/ Pten^tm1Hwu^ females. For studying the effects of PTEN knockdown on trachea function, pregnant females were injected intraperitoneally at the 14th day post coitum with 4 mg tamoxifen, dissolved in corn oil. The embryos were dissected and tracheas were isolated at day 19, before birth. To analyse the effect of PTEN knockdown on ciliation of ependymal cells, newborn pups were injected with 50 μg of tamoxifen. To reach higher knockdown efficiency, the injections were performed on 3 consecutive days (P0, P1 and P2). Ependymas were isolated for analysis at day 9 of postnatal development. For the analysis of BB translational polarity defects in ependymal cells, pups received three consecutive injections of 100 μg of tamoxifen starting at P3 and were dissected at P10. Since the crossing scheme results in multiple genotypes, PCR-based genotyping of embryos and pups was performed before further analysis. We used a set of primers to differentiate PTEN alleles previously published^23^, and the following primers to test for the presence of FOXJ1-Cre: 5′-TGACCGTACACCAAAATTTG-3′ and 5′-ATTGCCCCTGTTTCACTATC-3′. No blinding strategies were used for these experiments; however, all the littermates were treated and imaged under identical conditions. Sample sizes are indicated in the figure legends and are comparable to standards in the field.

### Cell culture and transfections

hTERT-RPE1 cells were purchased from ATCC (Cat# CRL-4000) and kept in DMEM:F12 (GIBCO/Invitrogen) with 10% fetal bovine serum (FBS) and 0.01 mg ml^−1^ of hygromycin B as recommended by ATCC. Cells were kept in culture for no more than 15 passages and routinely tested for mycoplasma. Cells were transfected with complementary DNA using PolyFect (Qiagen) or Lipofectamine LTX with Plus reagent (Invitrogen) and with siRNA at a final concentration of 50–60 nM, using Lipofectamine RNAiMax (Invitrogen) as per the manufacturer's instructions. The siRNA targeting human PTEN was a siGENOME SMART pool from Thermo Scientific (cat# M003023-02-0005) consisting of four different siRNAs with the following target sequences: D-003023-05, PTEN: 5′-GUGAAGAUCUUGACCAAUG-3′; D-003023-06, PTEN: 5′-GAUCAGCAUACACAAAUUA-3′; D-003023-07, PTEN: 5′-GGCGCUAUGUGUGUAUUAUUA-3′; D-003023-08, PTEN: 5′-GUAUAGAGCGUGCAGAUAA-3′. The sequence of control siRNA was 5′-GGGCAAGACGAGCGGGAAG-3′. To knockdown DVL2, STEALTH siRNA was used, targeting the 3′-untranslated region of human DVL2. The siRNA was custom-designed by Life Technologies and had the following sequence: 5′-CACAGUGGCCACAAUCUCCUGUAUG-3′ (antisense).

### Small-molecule inhibitors

To estimate the effect of inhibitors on the cilia disassembly process, the cells were starved for 40 h and then pre-incubated with the corresponding inhibitor for 2 h in starvation medium. Next, the DMEM/F12 medium containing 10% FBS and an inhibitor was added to induce the disassembly. The inhibitor was replenished at 7 h post serum addition for the 24 h time period. The drugs were used at the concentrations indicated in the figure legends.

We used CK1δ-ɛ inhibitor IC216 (Sigma, Cat#I0658), AURKA inhibitor MLN8237/Alisertib (Selleckchem, Cat#S1133), PLK-1 inhibitor BI2536 (Selleckchem, Cat#S1109) and PI3K inhibitor LY294002 (Cell Signalling, Cat#9901).

### Generation of hTERT-RPE1 stable cell lines

For preparing stable cell lines to rescue the PTEN knockdown-mediated enhanced cilia disassembly effect, cells were transfected with pCAGIP (empty vector) or human 3F-PTENr (PTEN RNA interference (RNAi)-resistant sequence) and then selected with puromycin at 10 μg ml^−1^. Single colonies were isolated, cultured and tested for 3F-PTENr expression and pAKT activation by western blotting (WB). The 3Flag-tagged PTEN resistant to RNAi (3F-PTENr) used in rescue experiments was designed by replacing 32 nucleotides from the human PTEN sequence (NM_000314) to introduce silent mutations (see Supplementary Methods). These mutations altered the sequences recognized by the four distinct siRNAs in siGENOME SMART pool for PTEN from Thermo Scientific (cat# M003023-02-0005). A triple-Flag-tag sequence was added at the N terminus of the PTEN RNAi-resistant sequence and was synthesized by GeneArt (Life Technologies). The final 3Flag-PTENr was subcloned into pCAGIP vector with puromycin resistance to generate stable lines in hTERT-RPE1 cells. For cell lines generated to test the role of S143 of DVL2, hTERT-RPE1 cells were transfected with pCAGIP (empty vector) or human 3F-DVL2 WT or S143A mutant and then selected with puromycin at 10 μg ml^−1^. Single colonies and pools were isolated, cultured and tested for DVL2 expression and pS143 signal by WB. Cilia disassembly experiments shown in Fig. 4 and related figures and images were conducted using pools from each construct.

### LUMIER screening

We performed a LUMIER screen^30^ looking for novel PTEN interactors using as bait a Renilla luciferase–PTEN fusion. The collection of Flag-tagged open-reading frames used for this screen contained 80 complementary DNAs, depicted in Fig. 3a. The screen was performed in duplicate (biological replicates), and positive interactions with luminescence intensity ratio values ≥3 in both screens were selected for confirmation by co-IP and western blot, using 3HA-tagged PTEN.

### Immunoprecipitation

To perform endogenous PTEN IP, six confluent 150-mm dishes of hTERT-RPE1 cells were rinsed with phosphate buffer, supplemented with 5 mM CaCl_2_ and 5 mM MgCl_2_ and lysed in 12 ml of hypotonic buffer containing 30 mM Tris-HCl pH 7.5, 5 mM CaCl_2_ and 5 mM MgCl_2_ and proteinase inhibitors, using a dounce homogenizer. Lysates were centrifuged at 500*g* and the supernatant was divided in two 6-ml aliquots to perform immmunoprecipitation with PTEN antibody and immunoglobulin (Ig)G control. The PTEN antibody (Cell Signaling Technology, cat# 9552S) was used at 1:300 dilution while 1.5 μg of Rabbit IgG (Sigma, cat # I5006) was used for the control. IP was performed for 3 h at 4 °C after which antibody complexes were precipitated by Protein G Dynabeads (Life Technologies, cat#10004D) for another hour. Samples were washed 5–6 times in the lysis buffer to reduce unspecific binding and the immune complexes were analysed by immunoblotting using PTEN and DVL2 antibodies combined with the Mouse anti-Rabbit Light-Chain-Specific HRP-coupled antibody (Jackson Lab, cat# 211-002-171).

### Immunoblotting

Unless otherwise indicated, lysis of HEK-293 T and hTERT-RPE1 cells was performed using TNTE 0.5% buffer (50 mM Tris-HCl pH 7.4, 150 mM NaCl, 1 mM tetrasodium EDTA, 0.5% Triton X-100) in the presence of protease inhibitors. Protein concentration was measured using the Pierce BCA Protein Assay Kit (Themo Scientific). For immunoblotting, protein lysates in Laemmli buffer were loaded on 8–12% SDS–polyacrylamide gels and then transferred to 0.45-μm nitrocellulose membrane (Bio-Rad, cat#1620115). Upon transfer, the membranes were blocked with 5% non-fat dry milk in Tris buffer saline containing 0.1% Tween (TBST) and incubated with primary antibodies overnight at 4 °C. Membranes were incubated with Goat Anti-Rabbit IgG coupled with horseradish peroxidase (1:10,000, GE Healthcare cat# RPN4301) or Sheep Anti-Mouse IgG coupled with horseradish peroxidase (1:10,000, GE Healthcare cat# RPN4301) and developed with SuperSignal West Dura, Femto (Thermo Scientific) or Clarity Western ECL Substrate (Bio-Rad). Images were acquired with Bio-Rad S-Max apparatus or Bio-Rad Chemidoc MP Imaging System. For publication purposes, original files were exported as 600 dpi tiff images, sporadically brightness and contrast settings were adjusted for the whole image without disturbing the image integrity (no ‘gamma' settings were changed). Due to similar protein mobility (DVL2 and pS143/DVL2; PTEN and AKT) samples from each experiment were run in different gels but processed simultaneously. Blots were generally cut along the 70-molecular-weight marker to probe the top part for pS143 or DVL2 and the bottom for PTEN, AKT or actin. The full-size blots from main figures are shown in Supplementary Fig. 11.

### Immunostaining and confocal microscopy

To perform immunostaining, cells and embryos were fixed for 10 min or 1 h with 4% PFA, respectively. The samples were permeabilized with 0.1% Triton X-100 and blocked in 3% BSA solution in phosphate buffer for 1 h at room temperature. For embryonic samples, the blocking solution was additionally supplemented with 0.1% Tween and 0.1% Fish Gelatin. Next, samples were incubated with correspondent primary and secondary antibodies, stained with 4′,6-diamidino-2-phenylindole and mounted into DABCO/Mowiol. In some cases, particularly for studying the localization of CLAMP-GFP and dDIX-Dsh-GFP, Triton X-100 permeabilization step was avoided, as it had an effect on CLAMP-GFP localization. The imaging was performed on a DMIRE2, Leica microscope with a spinning disk confocal scanner (CSU10, Yokogawa) connected to Hamamatsu EMCCD Digital camera (C9100-13). Volocity software (PerkinElmer) was used for image acquisition and analysis.

For analysis of cilia formation and disassembly in hTERT-RPE1 cells, the obtained raw data were exported as multi-page 16-bit tiff files without prior processing and analysed by Acapella high-content imaging and analysis software (PerkinElmer), as described below (see Cilia disassembly analysis). For analysis of MCCs (analysis of cilia, BB positioning and PTEN localization), images were acquired with either × 40/numerical aperture (NA) 1.25 or × 63/NA 1.32 objectives and then subjected to deconvolution using Volocity software with Point Spread Function (PSF) correspondent to the objective, iteration limit of 12 and confidence higher than 99%. Upon deconvolution, the brightness and contrast were adjusted using the same settings for all images, including control ones, within one library. For visualization of three-dimensional (3D) cilia models, images were generated with Volocity software. The analysis of rootlet polarity and study of dDIX-GFP localization were done using fluorescence signal of GFP and RFP tags, without subsequent staining with correspondent antibodies.

### Antibodies

The following antibodies were used to perform WB and immunofluorescence (IF) analysis. PTEN antibody (Cell Signaling Technology, cat# 9552S) dilutions for WB 1:2,000, for IF 1:300; acetylated α-tubulin antibody (Sigma-Aldrich, cat# T7451) 1:1,000 for IF; γ-tubulin antibody (Sigma-Aldrich, cat# T6557) 1:500 for IF; γ-tubulin antibody [TU-30] (Dyomics 647)(Abcam, ab#27076) 1:300 for IF; Pericentrin antibody (Abcam, cat# ab4448) 1:1,000 for IF; GFP antibody (Rockland, cat# 600-101-215) 1:500 for IF; RFP antibody (Abcam, cat# ab62341) 1:500 for IF; DVL 2 phospho-Serine-143 antibody (Abcam, cat# ab124933) 1:2,000 for WB; DVL2 antibody (Cell Signaling Technology, cat# 3224) 1:2,000 for WB; phospho-Akt (Ser473) (D9E) antibody (Cell Signaling Technology, cat #4060) 1:2,000 for WB; Akt antibody (Cell Signaling Technology cat#9272) 1:2,000 for WB; beta-actin antibody, clone AC-15 (Sigma, cat # A1978).

### Automated analysis of cilia disassembly in hTERT-RPE1 cells

For analysis of cilia assembly and disassembly, hTERT-RPE1 cells (1.05 × 10^6^) were plated on day 1 in 10-cm dishes and transfected with siRNAs on day 2 using Lipofectamine RNAiMax (Invitrogen) as per the manufacturer's instructions. At 48 h post transfection, cells were split and 20,000 were seeded onto round coverslips in 24-well plates for IF, and 120,000 cells in 12-well dishes for western blot. Three hours post split, cells were rinsed once and left in serum-free medium for 40–44 h to induce primary cilia formation. After starvation, cilia disassembly was induced by adding 10% of FBS and samples processed at each time point. Cells in 12-well dishes were lysed with TNTE buffer (50 mM Tris-HCl pH 7.4, 150 mM NaCl, 1 mM EDTA, 0.5% Triton, supplemented with protease inhibitors) at each time point and were processed for WB. Cells on coverslips were fixed and immunostained as described above. Images were acquired on Leica DMIRE2 microscope, equipped with Hamamatsu EMCCD camera, × 20/NA 0.5 objective, using Volocity software (PerkinElmer).

3D data sets (10–12 *z*-planes) were acquired for a minimum of 15 fields for each tested condition to capture at least 100 cells. The level of ciliation was assessed using a custom image analysis routine for Acapella high-content imaging and analysis software version 2.1 (PerkinElmer). Cilia were detected using a multi-step procedure: (1) 3D image data sets for pericentrin channel were maximum-intensity projected along *z* axis, followed by (2) detection of centrosomes; (3) 11 × 11-mm regions were cropped for each individual peri-centrosomal region from z-projected 3D images, acquired for acetylated α-tubulin channel, and used for (4) segmentation of bright clusters, representing a proximal part of a ciliary axoneme; (5) complete axonemal masks were detected using region growing method until contrast of a cilium was above a given threshold; (6) once ciliary boundaries had been segmented, the length of an axoneme has been measured (see Supplementary Fig. 4a). Image analysis script for Acapella version 2.1 and higher is available at GitHub^56^.

### Quantification of polarity defects in MCCs

For the analysis of rotational polarity defects in *Xenopus* MCCs, images of CLAMP-GFP and Centrin-RFP localization were captured using × 63/NA 1.32 objectives and then subjected to deconvolution using Volocity software (PerkinElmer) with Point Spread Function (PSF) correspondent to the objective, iteration limit of 12 and confidence higher than 99%. Next, the lines connecting basal rootlets with corresponding BBs were manually traced and the angles of basal rootlet orientation vectors (BROVs) were measured using Fiji^57^. Circular standard deviation (CSD) of BROVs was calculated for each cell. Each experimental condition was characterized by mean and s.d. of CSDs across all cells. To visualize the graphs summarizing rotational polarity defects, observed in different cells, the BROV angles were normalized by subtracting from their values the average angle of a correspondent cell, this difference was added to 90° and plotted on the graph (Fig. 1e). Code for MATLAB 2015a is available at GitHub^56^

For the analysis of translational polarity defects in EMCCs, we employed the algorithm previously published for the characterization of PCP defects in the same system^58^. In brief, ependymal cells, stained with Phalloidin, γ-tubulin and PTEN were imaged using the × 40/NA 1.25 objective and then exported as a tiff files using Volocity software (PerkinElmer). Next, cell borders were manually traced using Fiji^57^, based on F-actin label. Saved regions of interest were imported into MATLAB (MathWorks), followed by BB segmentation, based on adaptive thresholding within γ-tubulin channel. Centres of mass were calculated for BBs and cellular outline within every cell. Angles of BBOVs were measured and CSD across all BBOVs within every field of view was assessed using Circular Statistics Toolbox^59^. Each experimental condition was characterized by mean and s.d. of CSDs across all fields of view. Similar to basal rootlet polarization measurement, CSD values were calculated for BBOV angles. The angles measured within one image plane were normalized by subtracting from their values the average angle of a corresponding field of view, this difference was added to 90° and plotted on the summarized graph (Fig. 2c,d). In total, for WT condition we analysed 669 ependymal cells from 8 animals; for heterozygous littermates, we evaluated 292 cells from 4 different animals; for Pten conditional knockout, we evaluated 727 cells with visible Pten knockdown from 11 different pups. Statistical analysis of circular data was done using Circular Statistics Toolbox^59^ for MATLAB (MathWorks). Angle histograms were displayed using rose plot in MATLAB (MathWorks). Code for MATLAB 2015a is available at GitHub^56^.

### Code availability

The automated image analysis was performed as described in the sections ‘Automated analysis of cilia disassembly in hTERT-RPE1 cells' and ‘Quantification of polarity defects in MCCs'. Codes are available through GitHub following the link: https://github.com/LTRI-HCS/PTEN-paper, as cited above.

### Phosphopeptide and protein phosphatase activity assay

*N*-terminally biotinylated phosphoserine peptides (10 μM), custom-made by Genscript (GGSFHPNVSS(pSER)HENLEPE ((pSER)—phosphorylated Serine)) in blocking buffer: 50 mM Tris pH 7.5, 150 mM NaCl, 0.1% Tween 20 (TBST) with 1% (w/v) BSA were immobilized on a streptavidin-coated strip plate (Millipore) for 1 h at 37 °C. After washing, the wells were blocked for 2 h with blocking buffer before incubation with recombinant PTEN, obtained using baculoviral expression system (see Supplementary Methods for details) in phosphatase buffer: 10 mM HEPES pH 7.5, 1 mM EGTA and 10 mM dithiothreitol. After 10 min of incubation at 37 °C, wells were washed thoroughly with TBST, before a final 10-min wash with blocking buffer. Wells were then incubated with anti-phospho-serine-143 antibodies for 3 h at 25 °C. The plate was then washed 10 times with TBST, and then incubated with Anti-Rabbit IgG HRP-linked Antibody (Cell Signaling Technologies), diluted 1: 75,000 in blocking buffer. After washing with TBST, and a final wash with distilled water, the TMB substrate reagent (Sigma) was added and the reaction allowed to proceed until negative controls showed coloration, whereupon the reaction was quenched with 2 M sulphuric acid. The absorbance at 450 and 570 nm was measured, and the percentage substrate turnover calculated from control experiments. In addition, phosphoserine polypeptide in the concentration of 10 μM was incubated with equimolar amounts of PTEN for 1 h at 37 °C in the buffer containing 0.1 M Hepes pH 7.4, 100 mM NaCl, 10 mM MgCl_2_ and 1 mM MnCl_2_ and then the reaction was stopped by addition of 8% Trifluoroacetic acid (TFA) and analysed by mass spectrometry on LTQ-XL (Thermo Scientific). To demonstrate the PTEN-specific nature of the reaction, we used PTEN pre-incubated for 5 min with the specific inhibitor VOOH-pic (cat#V8639, Sigma), at a concentration of 1 μM. We used the pSerine-143 peptide without protein as a negative control.

To assess protein phosphatase activity of PTEN against full-length DVL2, we used baculovirally expressed PTEN at final concentration of 0.1 mg ml^−1^ and incubated it with 3Flag-tagged DVL2 in the assay buffer, containing 30 mM TrisCl, pH 7.5, 10 mM MgCl_2_ and 10 mM dithiothreitol, reaction volume 50 μl. As a control of unspecific phosphatase activity, we used same amounts of PTEN C124S, an inactive mutant of PTEN as well as PTEN 6A, which is a constitutively active mutant, in which serines 370, 380, 385 and threonines 366, 382, 383 were mutated to alanine residues. 3F-DVL2 WT as well as its phosphorylation-dead construct, where serine 143 was mutated into alanine, were overexpressed in HEK293T cells. To purify DVL2 proteins, cells were lysed in the buffer containing 20 mM TrisCl, pH 7.5, 150 mM NaCl, 1 mM EDTA and 0.5% Triton X-100, and then IP was performed using Anti-FLAG M2 Magnetic Beads (Sigma, cat# M8823). After 4 h of IP, the beads were washed with lysis buffer and then the buffer was exchanged to the assay buffer, in which DVL2 proteins were eluted by the addition of 100 μg ml^−1^ of Flag peptide (Sigma, cat# F4799) in a total volume of 100 μl. Next, half of eluate was incubated with recombinant PTEN, while the other half was used as a control.

## Additional information

**How to cite this article:** Shnitsar, I. *et al.* PTEN regulates cilia through Dishevelled. *Nat. Commun.* 6:8388 doi: 10.1038/ncomms9388 (2015).

## Supplementary Material

Supplementary Information Supplementary Figures 1-11, Supplementary Methods and Supplementary References

Supplementary Movie 1 Flow of fluorescent beads at dorsolateral surface of an embryo injected with control morpholino. The movie was recorded at 30 fps and played at 10 fps. Note that bead flow is highly organized and unidirectional from anterior side (on the right) to posterior side (on the left)

Supplementary Movie 2 Two examples of fluorescent beads flow at dorsolateral surface of embryos, injected with PTEN morpholino. The movie parameters are the same as for Movie 1. Note that Bead flow is distorted and slower compared to control.

Supplementary Movie 3 Two examples of fluorescent beads flow at dorsolateral surface of embryos, injected with PTEN morpholino. The movie parameters are the same as for Movie 1. Note that Bead flow is distorted and slower compared to control.

Supplementary Movie 4 Flow of fluorescent beads at dorsolateral surface of an embryo co-injected with PTEN morpholino and human HA-tagged PTEN. The velocity, as well as directionality, of the bead flow is restored
