# Supplementary material for: PTEN regulates cilia through Dishevelled
Source: Nat Commun. 2015 Sep 24;6:8388. doi: 10.1038/ncomms9388 (PMC4598566; doi:10.1038/ncomms9388)
Supplement: Supplementary Information — Supplementary Figures 1-11, Supplementary Methods and Supplementary References [file ncomms9388-s1.pdf]

Supplementary Figures

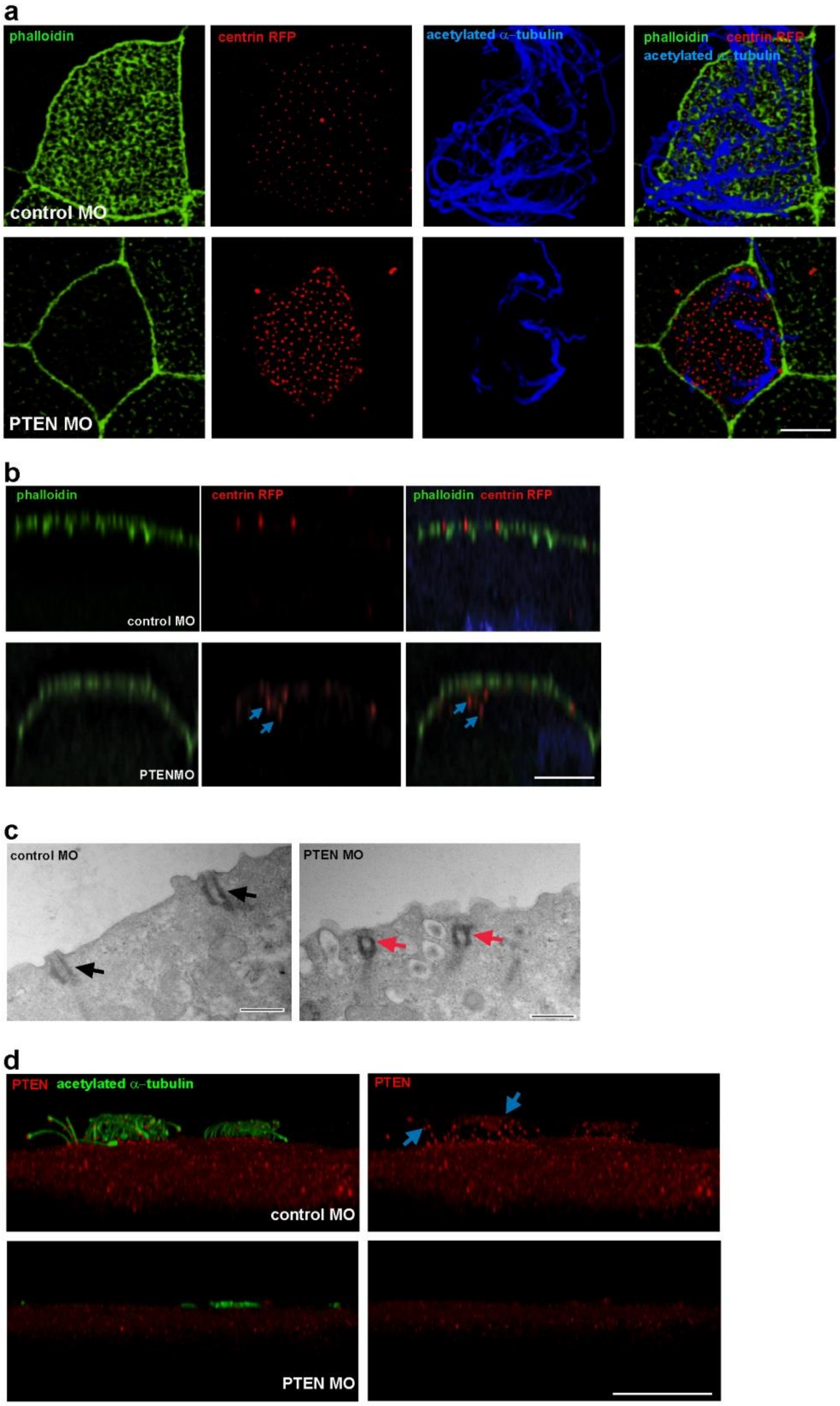

Supplementary Figure 1. PTEN knockdown affects cilia formation in *Xenopus* multi-ciliated cells. (**a**) Epidermis of embryos treated with control MO or PTEN MO, showing defects in multi-cilia (bottom panel). Cilia axonemes are visualized by acetylated α-tubulin staining

(blue), cortical F-actin is stained with fluorescent phalloidin (green) and cilia basal bodies (BB) are labelled with RFP-centrin (red) (scale bar is 7 μm), n=6. (**b**) Loss of PTEN function results in BB apical docking defects. Apical membrane was labeled with phalloidin (green), BBs were labeled with RFP-centrin (red) in both control MO and PTEN MO treated embryos. Apical-basal reconstruction of confocal images shows BB docking defects in PTEN morphants compared to control (scale bar is 7 μm), n=4. (**c**) Transmission electron microscopy demonstrates abnormal BB docking (arrows) in PTEN morphant embryos (PTEN MO, right panel, red arrows) compared to control morpholino injected embryos (control MO, normally positioned BB, black arrows), n=2. (**d**) PTEN localizes to cilia axoneme. Endogenous staining for PTEN (red) shows its ubiquitous presence in multi-ciliated cells with enrichment on the apical side. Note the spotted pattern for PTEN, localized in cilia axoneme, labeled with acetylated α-tubulin (green) and its absence in PTEN morphants (PTEN MO). Scale bar is 20 μm.

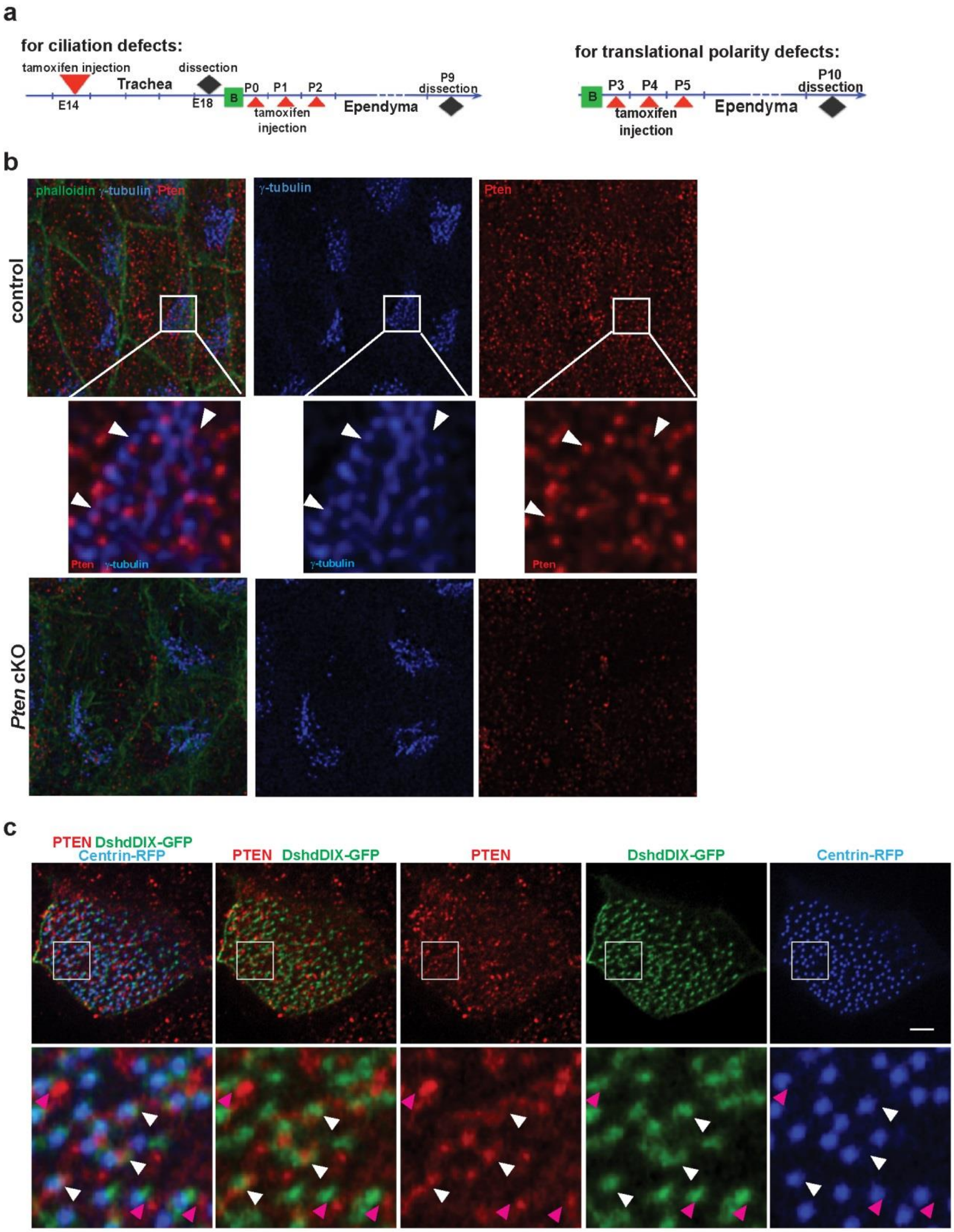

Supplementary Figure 2. Analysis of *Pten* loss in mouse ependymal cells. (**a**) Two tamoxifen induction schemes used in Figure 2 experiments. (**b**) Ependyma apical surface 3D reconstruction showing Pten localization at the basal body area, enlarged at the middle bottom panel. Examples of Pten localized in close proximity to basal bodies are marked with white arrows. The lower

panel shows ependyma of a conditional knockout littermate (*Pten* cKO), stained with phalloidin, gamma-tubulin and Pten antibodies to confirm specificity of the staining. Scale bar is 10 μm. (**c**) Confocal section of *Xenopus* multi-ciliated cell, showing the co-localization of endogenous PTEN with Dishevelled, lacking DIX domain. PTEN is labelled in red, the localization of dDIX-Dsh-GFP was observed via GFP fluorescence (green), basal bodies were labelled by RFP-Centrin (blue). Note that both PTEN and dDIX-Dsh-GFP signals are found in the area close to basal bodies. Interestingly, the proteins display 2 different types of localization: at some points they co-localize with each other (white arrows at the lower panel), while at some areas they are found adjacently (magenta arrows). Scale bar is 3 μm.

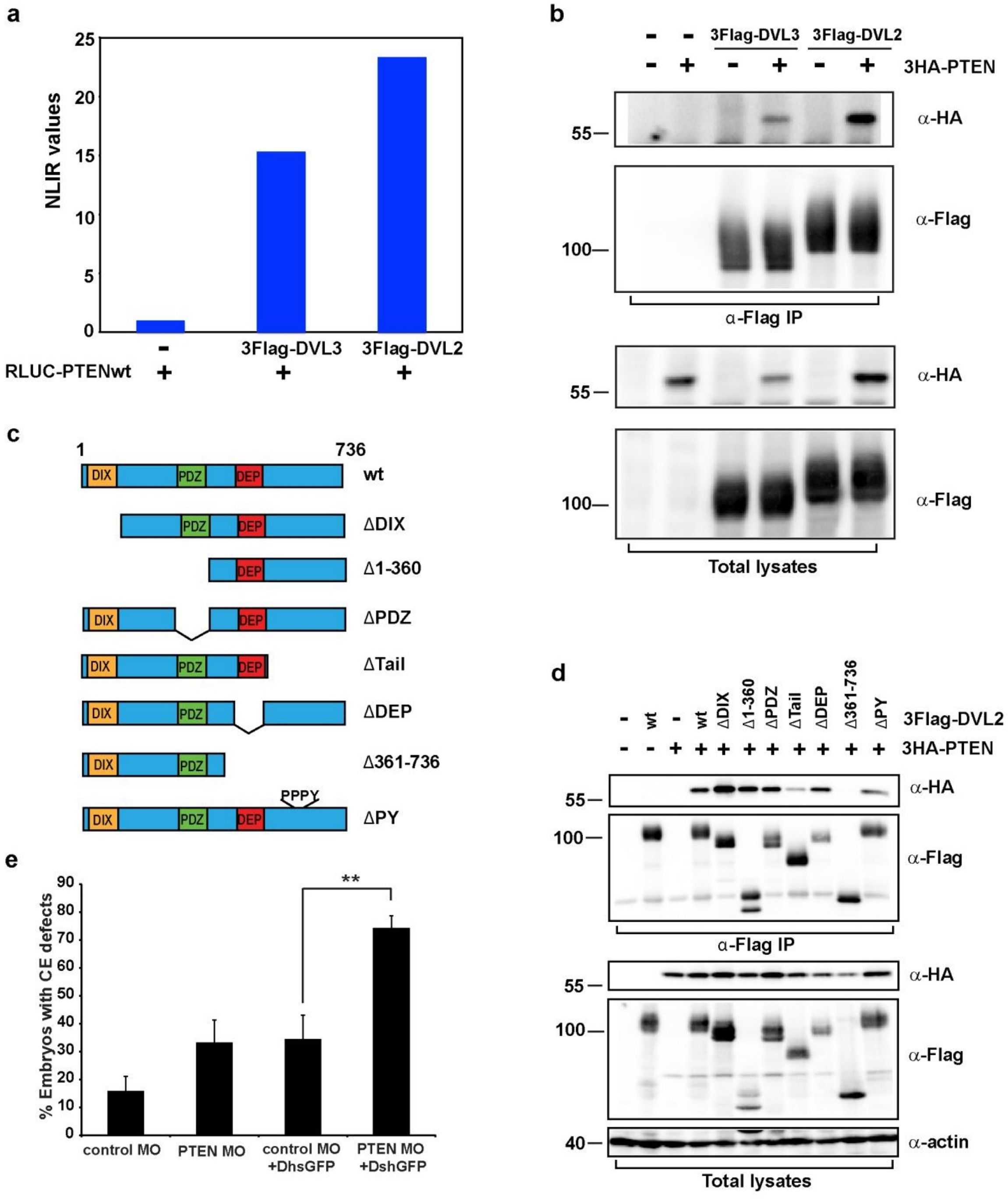

Supplementary Figure 3. Confirmation of the interaction between PTEN and Dishevelled proteins. (**a**) Manual LUMIER assay. HEK293T cells were transfected with RLUC-PTEN alone or with 3Flag-DVL2 or 3Flag-DVL3. Lysates were subjected to anti-Flag immunoprecipitation and Renilla luciferase activity was measured in the immune-complexes. The NLIR values in the y axis, indicate the LIR values normalized to the expression levels of RLUC-PTEN in total lysates. Data is average from 2 technical replicates (n=1). (**b**) 3HA-PTEN was co-transfected

with 3Flag-DVL2 or 3Flag-DVL3 in HEK-293T cells. After immunoprecipitation using anti-Flag antibody, samples were immunoblotted using the indicated antibodies. Immunoblots are representative from 3 independent experiments. (**c**) DVL2 mutants used to identify protein-protein interaction motifs between PTEN and DVL2. (**d**) Mapping of the PTEN interaction domain in DVL2. 3HA-PTEN was transfected in HEK293T cells alone or together with WT or mutant 3F-DVL2 harboring different domain deletions indicated in the schematic on **c**. Lysates were processed for anti-Flag immunoprecipitation as in **b**. Immunoblots are representative of 3 independent experiments. (**e**) PTEN Functions in Convergent Extension (CE) morphogenetic movements. *Xenopus* embryos injected with control or PTEN morpholinos (PTEN MO) either alone or together with low doses (100 pg) of WT Dishevelled (DshGFP) as indicated, were then scored for CE defects. The graph displays the mean with error bars representing s.d. with 100 and more embryos counted for each condition (**$p<0.01$ by t test; n=3). Note that interference with PTEN strongly synergizes with DshGFP to induce CE defects.

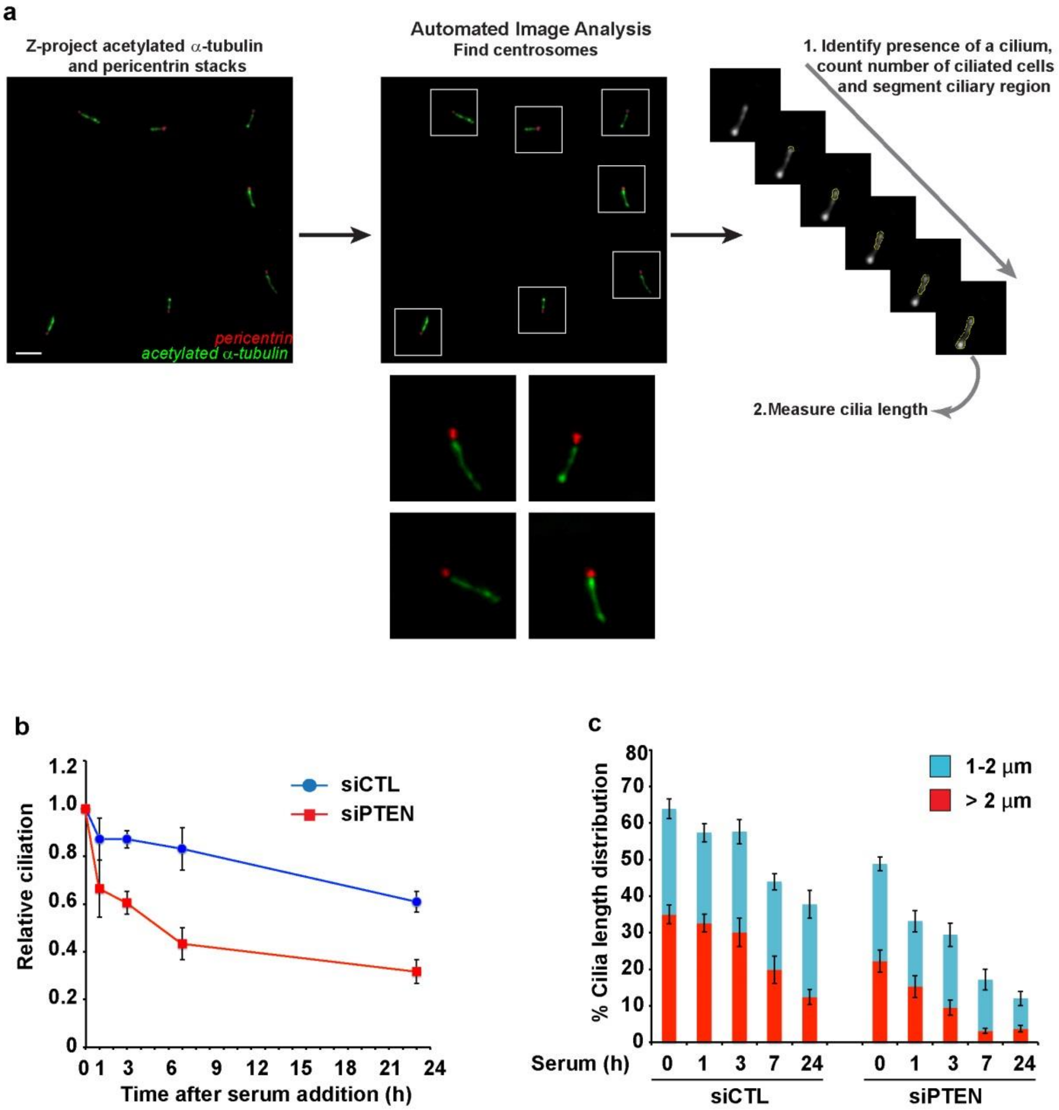

Supplementary Figure 4. PTEN knockdown increases cilia disassembly rate. (**a**) Schematic diagram of the custom image analysis algorithm for assessment of ciliation and morphological measurements. Z-stacks of acetylated α-tubulin-stained axonemes (green channel) and pericentrin-stained basal bodies (red channel) were maximum-intensity projected. Basal bodies were located using pericentrin image, followed by crop of a peri-centrosomal region to test presence of a cilium, originating from a given basal body. Axonemes were segmented using region growing method and ciliary length was measured (scale bar is 5 μm). (**b**) hTERT-RPE1 cells were transfected with small interfering RNAi control (siCTL) or PTEN (siPTEN) as described for **Figure 4b**. Forty-eight hours post-transfection cells were starved for 40 h more to induce cilia formation. Serum was added to trigger cilia disassembly and cells were fixed at the

indicated time points (x axis). Cells were immunostained for cilia axonemes with anti-acetylated α-tubulin and for basal bodies with anti-pericentrin antibodies. The ciliation in each condition was evaluated using Acapella customized routine as described in **a**, then normalized to the percentage of ciliated cells at time 0 h of serum and plotted in the y axis. Data points display the mean with error bars showing s.d. (n=7; n=4 for 7h time point). At least 1000 cells were counted for each condition. (**c**) Cilia length was determined for each condition in **b** using the custom image analysis routine and segregated in 2 categories, 1 to 2 μm long and longer than 2 μm. The percentage of cilia with averaged specified length (error bars represent s.d.), is shown at the y axis for each time point after serum addition.

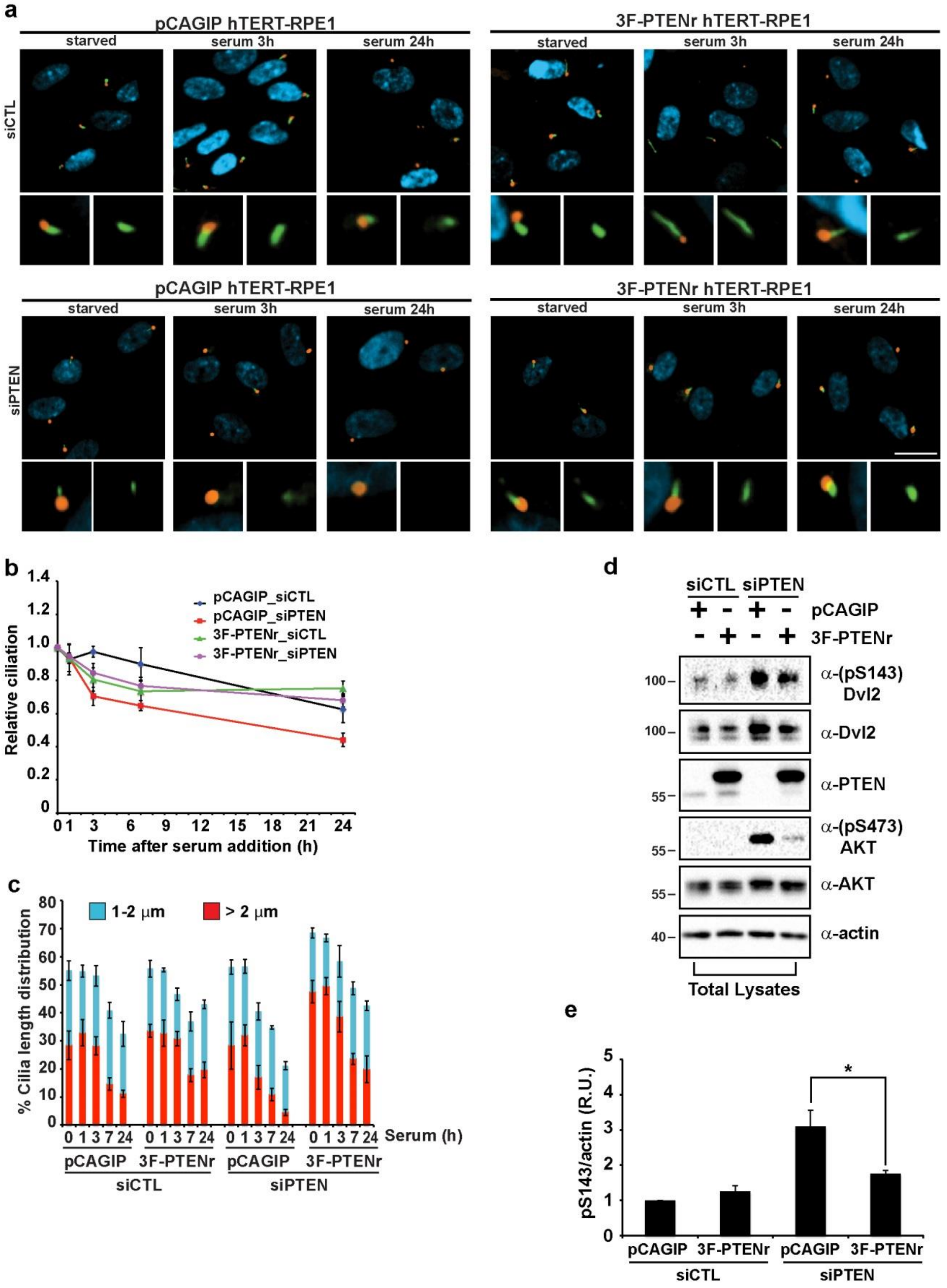

Supplementary Figure 5. Increased cilia disassembly rate upon PTEN knockdown is rescued by RNAi-resistant PTEN. (**a**) hTERT-RPE1 cells stably expressing an empty vector

(pCAGIP) or a RNAi-resistant 3Flag-tagged PTEN (3F-PTENr) were transfected with siCTL or siPTEN as described in **Figure 4f**. After 48 h post-transfection, cells were starved for 40 h to promote cilia formation. To induce cilia disassembly, serum was added and cells were subjected to analysis at several time points. Representative images of cells are shown under the specified conditions fixed at 0 h (starved), 3 h and 24 h post-serum addition. Cells were immunostained for cilia axonemes with anti-acetylated α-tubulin (green) and for basal bodies with anti-pericentrin (red) antibodies. Cell nuclei were stained with DAPI (blue) (scale bar is 20 μm). (**b**) Cilia numbers from stable hTERT-RPE1 cell lines in **a** were quantified from each condition at 0, 1, 3, 7 and 24 h after serum addition and normalized to the cilia number at 0 h from siCTL-transfected pCAGIP expressing cells and plotted in the y axis. Graph shows the mean with error bars representing s.d. (n=4). (**c**) Cilia length was evaluated using the Acapella customized routine and categorized in 2 groups from each condition in **b** as indicated. Percentage of cilia with the averaged specified length is shown in the y axis (error bars indicate s.d.; n=4). (**d**) pS143-DVL2 levels are lower in cells expressing 3F-PTENr. Stable hTERT-RPE1 cell lines expressing pCAGIP or 3F-PTENr were transfected and treated as in **a**. After 40 h of starvation (0 h serum) cells were lysed and analyzed by immunoblotting using the indicated antibodies. Blots are representative from 5 independent experiments. (**e**) Quantitative analysis of Serine-143 DVL2 phosphorylation from experiments in **d** was performed using Image Lab 4.1 software (Bio-Rad). The intensity values of pS143-DVL2 in the indicated conditions were normalized by corresponding values of actin intensities. Generated ratios were subsequently normalized by the value obtained in siCTL-treated pCAGIP-expressing cells and plotted as Relative Units (R.U.) in the y axis (error bars indicate s.e.m.; *$p<0.05$ by t test; n=5).

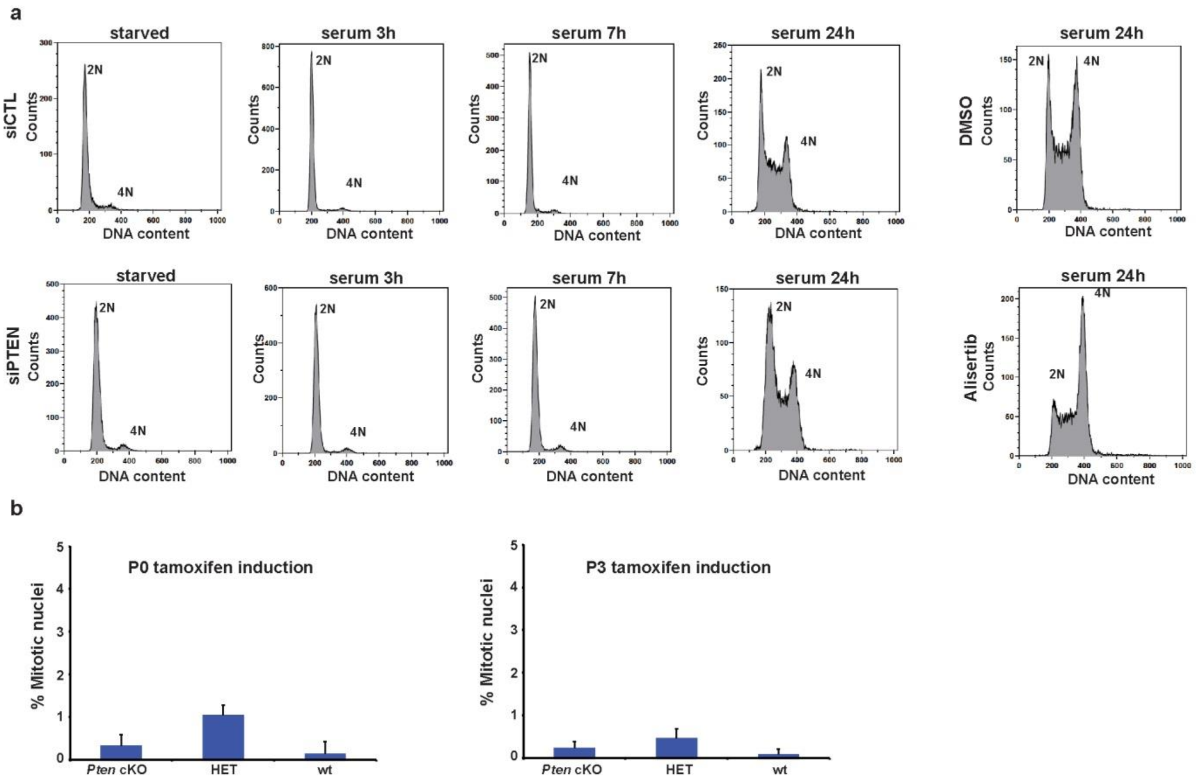

Supplementary Figure 6. PTEN knockdown does not promote early cell cycle re-entry during cilia disassembly. (**a**) hTERT-RPE1 cells were transfected with siCTL or siPTEN and treated as in **Figure 4b** for cilia disassembly. Cells were fixed at the time points indicated after serum addition, stained with Propidium iodide and subjected to FACS analysis. In parallel and as control to detect cell cycle entry inhibition, cells were treated with Alisertib (5 μM) or DMSO 2 h prior serum stimulation. DMSO and Alisertib were replenished at the time of serum addition and again after 7 h of serum. DNA content is displayed in the X-axis (2N diploid; 4N tetraploid) and cell numbers in Y-axis. Data shown is representative from 4 independent experiments. (**b**) Percentage of mitotic nuclei in mouse ependymal cells was quantified via manual counting of mitotic nuclei stained with DAPI. The quantification was done on ependymal slices, obtained from 2 groups of littermate pups. Group1 (graph on the left) was treated with tamoxifen on P0-P2 and analyzed at P9, whereas Group2 (graph on the right) received tamoxifen injections on P3-5 and was analyzed at P10. Abbreviation "WT" denotes $Pten^{Hwu}FOXJ1\text{-}Cre^{-}$, "HET" is $Pten^{Hwu/WT}FOXJ1\text{-}Cre^{+}$ and "cKO" is $Pten^{Hwu/Hwu}FOXJ1\text{-}Cre^{+}$. The numbers of nuclei analyzed during the experiments were: Group1 WT, 2921 nuclei; HET, 723 nuclei; cKO, 5121 nuclei. Group2 WT, 831 nuclei; HET, 1773 nuclei, cKO, 3670 nuclei.

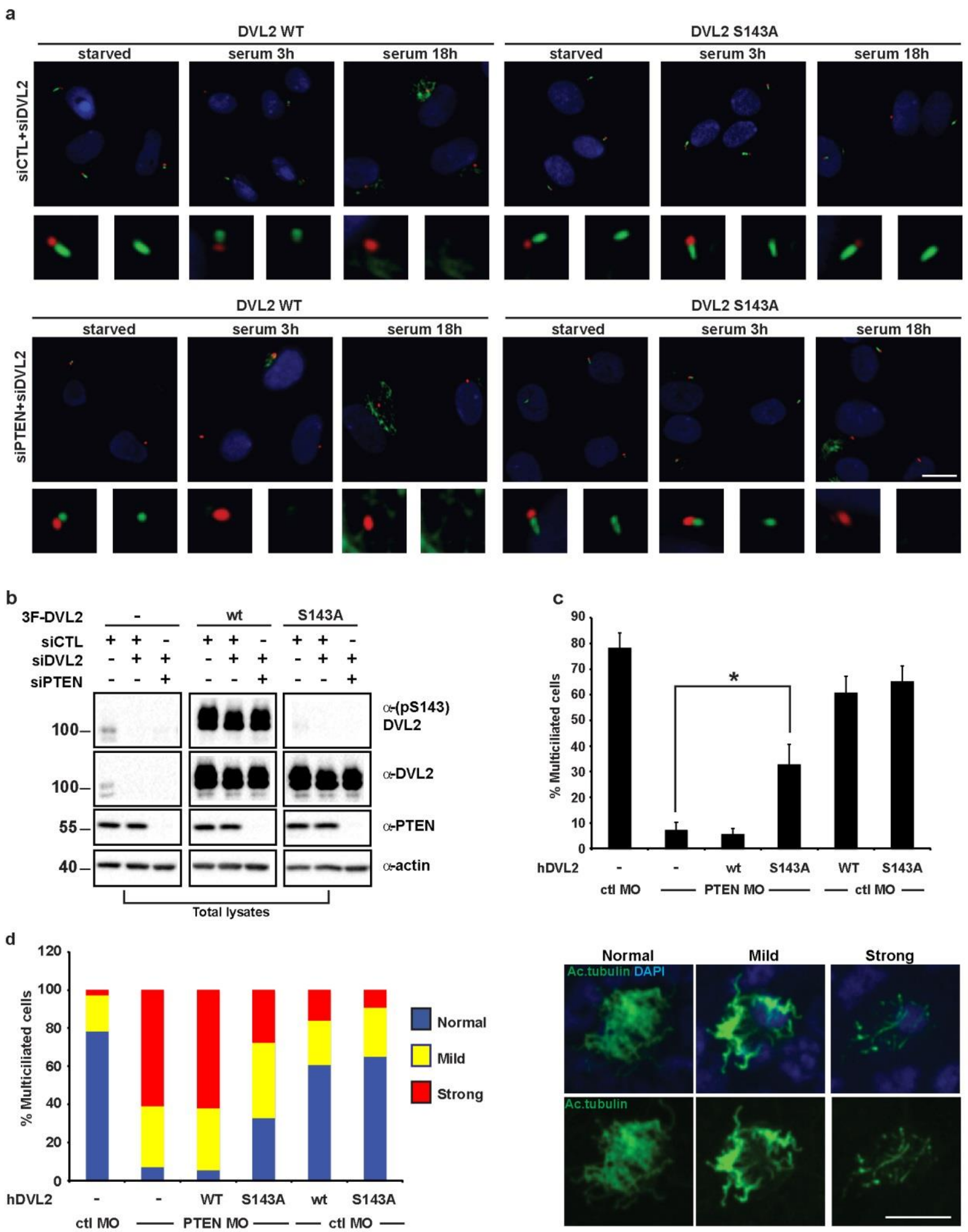

Supplementary Figure 7. S143 is a crucial residue mediating PTEN/DVL2-dependent cilia disassembly. (**a**) Representative images of cilia from a disassembly assay upon serum stimulation in hTERT-RPE1 stable lines, expressing 3F-DVL2 WT or its S143A phosphorylation mutant and transfected with siRNAs as indicated. DVL2 siRNA targets its endogenous 3’-UTR. To visualize ciliation, cells were stained for acetylated tubulin (green), pericentrin (red) and

DAPI (blue) at the indicated conditions and time points. Note that cilia rapidly disassemble when DVL2 WT is expressed, while cilia are stabilized by expression of DVL2 S143A (scale bar is 20 μm). (**b**) Cells treated as in **a** were lysed after 40 h starvation. Equal amounts of protein were immunoblotted with antibodies recognizing (pS143)-DVL2, DVL2 and PTEN to confirm the expression of constructs and knockdown efficiency. Blots are representative of samples from experiments in **Figure 4g** (n=4). (**c**) *Xenopus* embryos were injected with control (ctl) or PTEN morpholinos (MO) with or without human DVL2 WT or S143A mutant. Multi-ciliated cells on tadpoles skin were quantified by staining axonemes with acetylated tubulin. The percentage of normal multi-ciliated cells was plotted as mean with error bars showing s.e.m. of n=5; *$p<0.05$ by t test. An average of 130 cells were analyzed per condition. (**d**) The effects on multi-ciliated cells analyzed in **c** were segregated as normal, mild or strong according to the observed acetylated tubulin staining (right panel) and plotted. Scale bar is 10 μm.

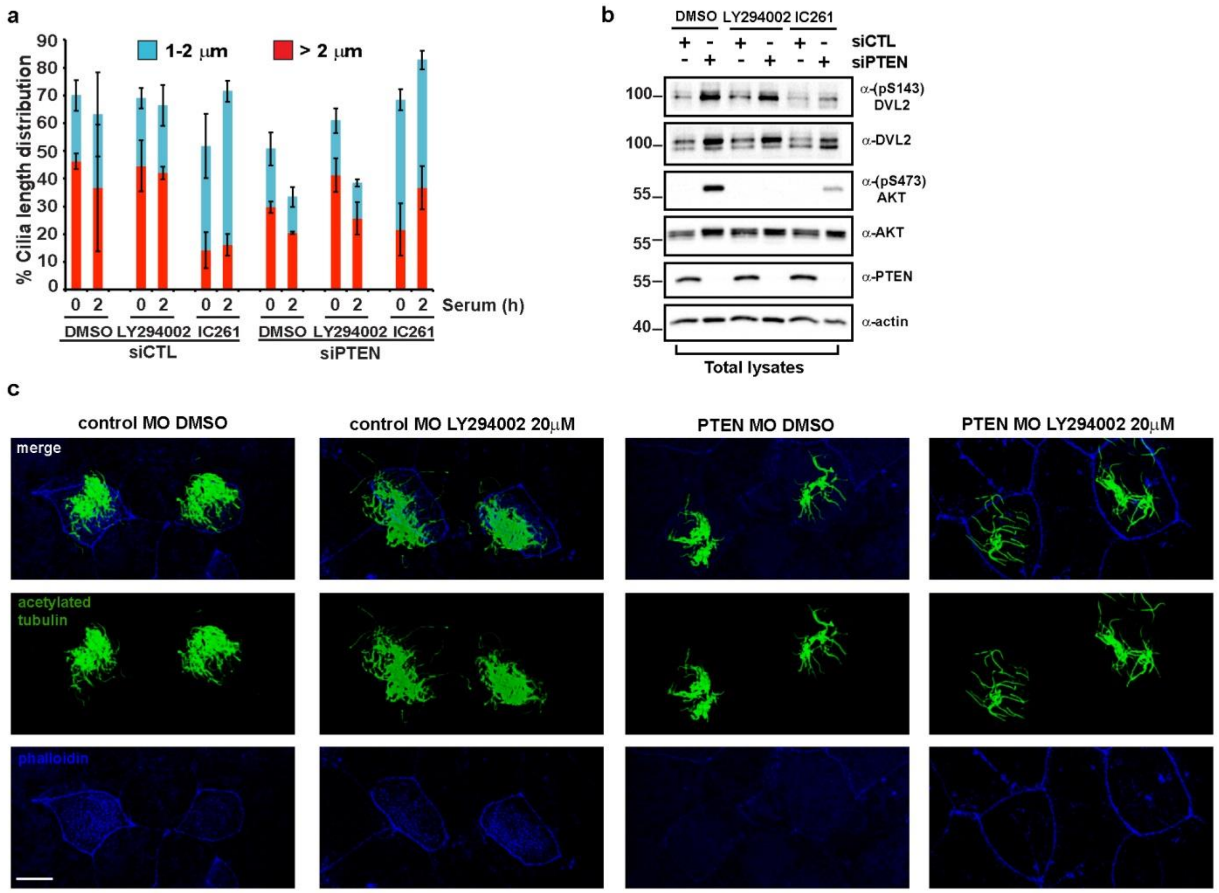

Supplementary Figure 8. PTEN regulates cilia in a PI3K-independent manner. (**a**) Cilia length distribution in the presence of PI3K and CK1ε inhibitors. Cilia length was quantified from hTERT-RPE1 cells in **Figures 5a-b**. The graph shows averages with error bars representing s.d., n=3. (**b**) IC261 but not LY294002 treatment blocks the enhanced pS143S caused by PTEN knockdown in hTERT-RPE1. Cells were transfected with siCTL or siPTEN, starved and treated with the inhibitors for 2 h and lysed before serum addition. Cell lysates were immunoblotted with the indicated antibodies and are representative from n=3 in **Figure 5a**. (**c**) *Xenopus* epidermis, stained with acetylated α-tubulin (axonemes, green), and phalloidin (blue), from embryos treated with 20 μM LY294002 or DMSO. Axonemes showed cilia number reduction in PTEN morphants independent of PI3K inhibition by LY294002. Embryos were treated with LY294002 as they completed gastrulation (NF12.5) and grown until tadpole stage. No gross morphological defects or increased mortality was observed upon LY294002 treatment (scale bar is 10 μm). Images are representative from 2 biological replicates.

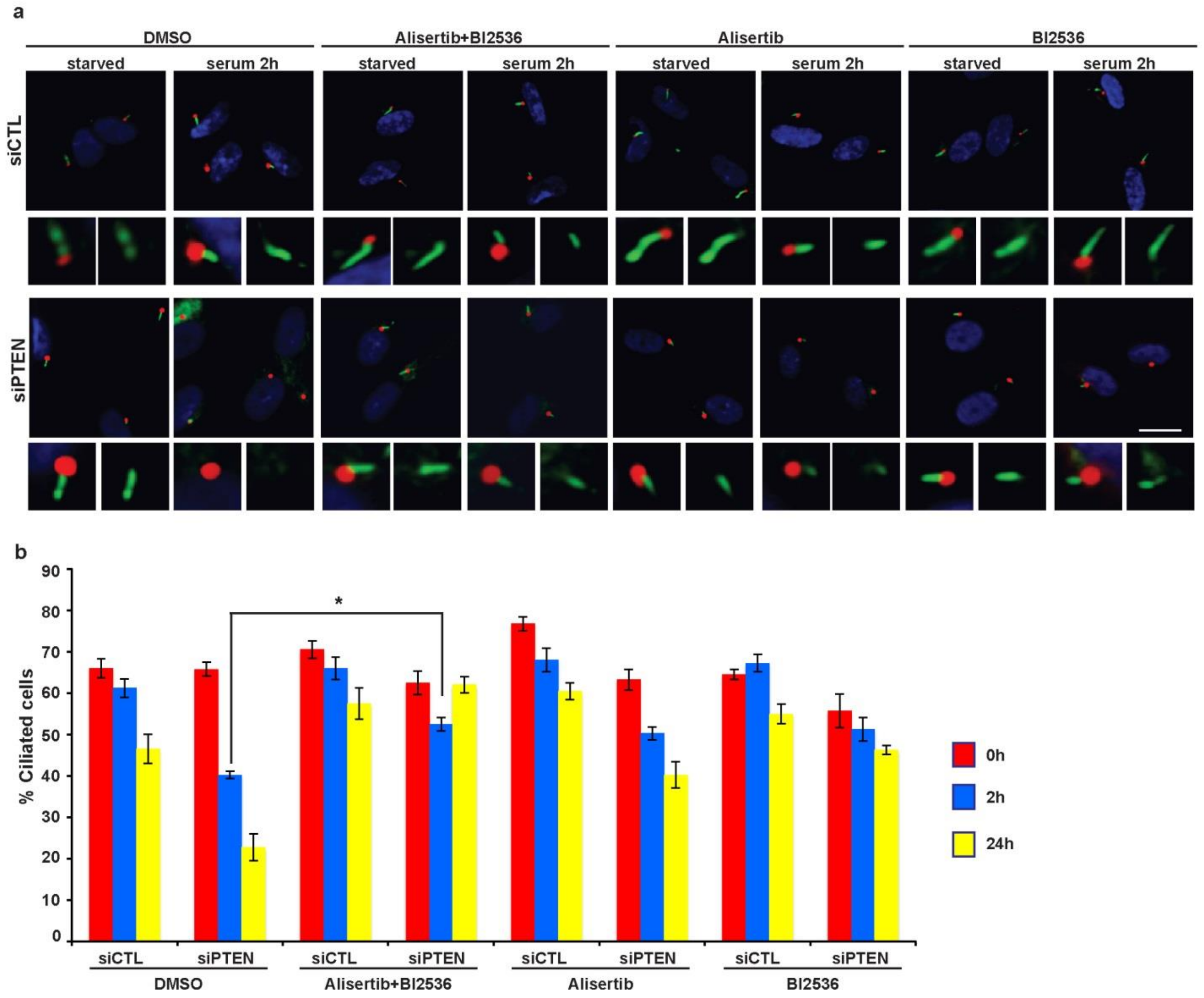

Supplementary Figure 9. Aurora kinase A and Polo-like kinase 1 are involved in cilia disassembly driven by PTEN. (**a**) Representative images of cilia from hTERT-RPE1 cells transfected with siCTL and siPTEN during cilia disassembly assays in the presence of Aurora kinase A (AURKA) or Polo-like kinase 1 (PLK1) inhibitors. Percentage of ciliated cells was analyzed at the indicated time points: starved (before serum addition), 2 h and 24 h after serum stimulation. For ciliation analysis cells were stained for acetylated tubulin (axonemes, green), pericentrin (centrioles, red) and DAPI (nuclei, blue). The PLK1 inhibitor BI2536 was used at 1 μM. The AURKA inhibitor Alisertib was used at 10 μM by itself and at 5 μM in combination with BI2536 (scale bar is 20 μm). (**b**) Ciliation of hTERT-RPE1 cells was quantified using the custom image analysis routine described in **Supplementary Figure 4a** and displayed as percentage of ciliated cells (mean with error bars showing s.e.m. of n=4; *p<0.05 by t test).

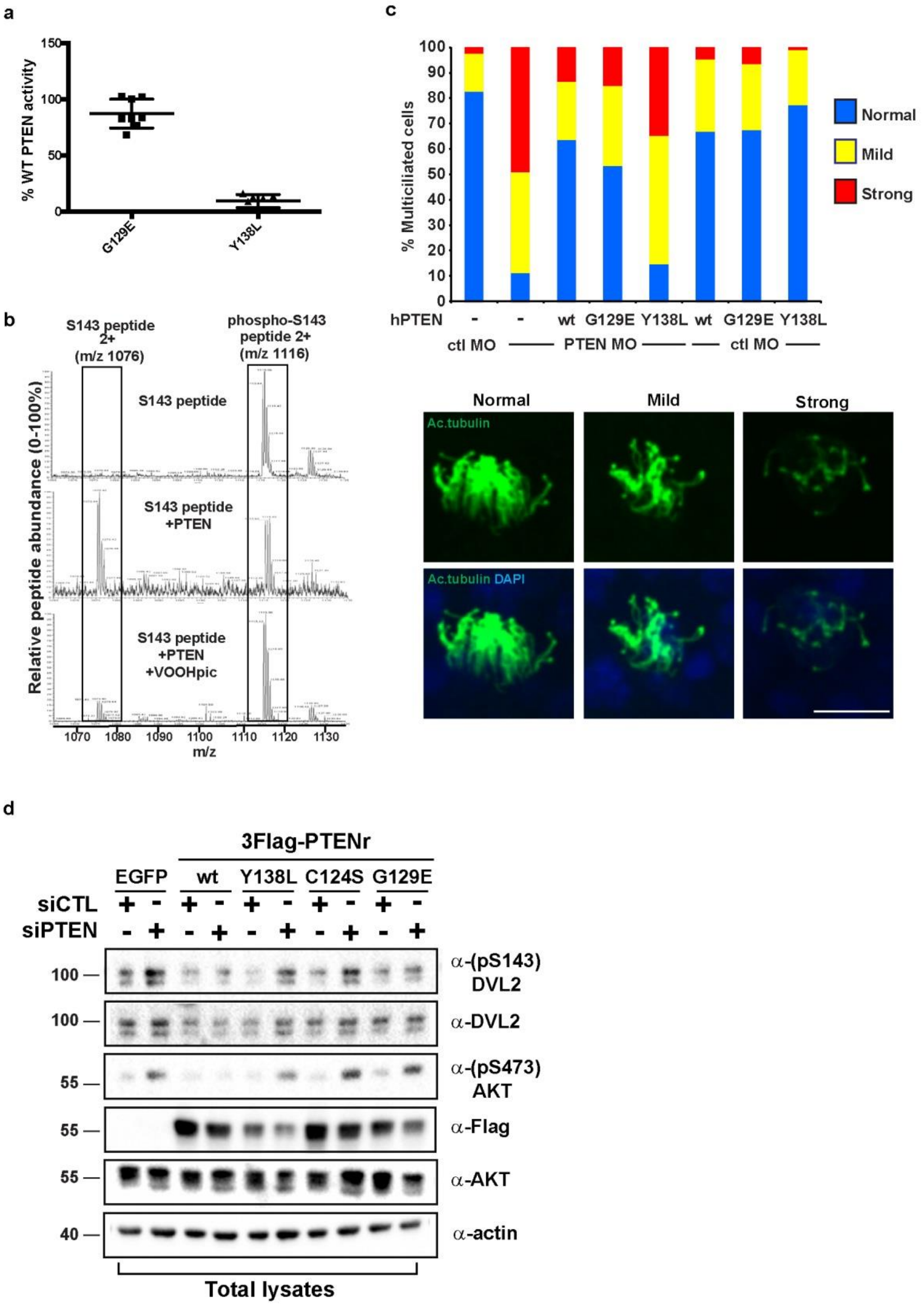

Supplementary Figure 10. PTEN requires its protein but not lipid phosphatase activity to regulate DVL2 Serine-143 phosphorylation and multi-cilia in *Xenopus* epidermis. (a)

PTEN WT, G129E and Y138L purified from baculovirus system were incubated with the pS143 DVL2 peptide for 30 minutes and percentage of remaining pSerine was evaluated using the specific anti-pS143 DVL2 antibody in an ELISA-type assay. A PTEN-free well and a well with non-phosphorylated peptide were used as controls. Results are plotted as percentage of the activity observed with PTEN WT protein. Graph shows mean with error bars indicating s.d. Experiments were carried out in triplicate and repeated 3 times. (**b**) Specificity of the Ser-143 dephosphorylation by PTEN. Mass-spectrometry analysis of the reaction between PTEN purified from baculovirus system and the pSerine-143 peptide of DVL2 (**Figure 5 d-e**). The presence of a 2+ peak, corresponding to the dephosphorylated form of the peptide is evident in the condition containing active PTEN (middle panel, left column) and it can be reduced by the pre-treatment of equimolar amounts of VOOH-pic, a PTEN-specific inhibitor (bottom panel, left column). Figure is representative of 3 independent experiments. (**c**) Effects on multi-ciliated cells in *Xenopus* analyzed from **Figure 5g** were segregated and plotted into 3 groups: normal, mild or strong (upper panel), according to the observed acetylated tubulin staining (lower panel). Scale bar is 10 μ□□ (**d**) Overexpression of lipid phosphatase inactive PTEN G129E mutant rescues the increased pS143-DVL2 levels caused by PTEN knockdown, while protein phosphatase inactive PTEN Y138L mutant does not. hTERT-RPE1 cells were transfected with siCTL or siPTEN and then transduced with lentiviruses expressing Flag-tagged RNAi resistant PTEN (3Flag-PTENr) wild type (WT) or mutants. Cells were subsequently starved for 48 h, lysed and processed for immunoblotting to evaluate pS143-DVL2. Note that PTEN Y138L downregulates the levels of pS473 on AKT less efficiently compared to PTEN WT. In similar experiments using U-87 MG cells, PTEN mutants displayed the expected behaviour towards pS473-AKT as previously reported[1], suggesting that the incomplete rescue of pS473 AKT by PTEN Y138L is cell-type specific. This figure is representative of 3 independent experiments.

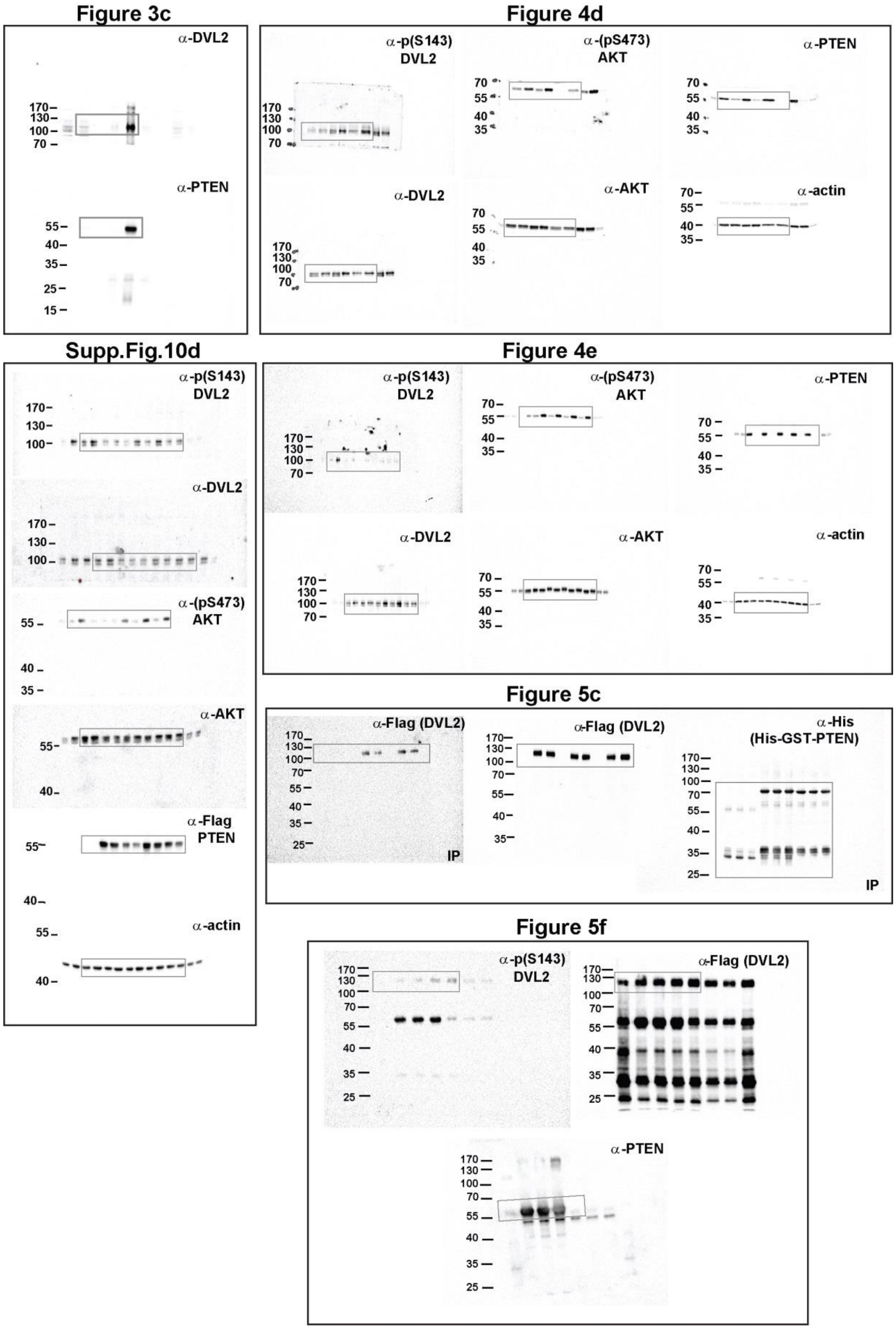

Supplementary Figure 11. Full-size scans of immunoblots presented in the Main Figures. Corresponding figures are indicated above each rectangle.

Supplementary Methods

Generation of PTEN constructs and mutants

The human 3Flag-PTEN catalytically dead mutant (C124S), the lipid phosphatase mutant (G129E) and the protein phosphatase mutant (Y138L) were generated using the QuikChange site-directed mutagenesis kit from Agilent in a pCMV5 vector using the 3Flag-PTENr sequence as template. The primers used for C124S were:

5'-CATGTTGCAGCAATTCACTCAAAAGCTGGAAAGGGACGAA-3' and

5'-TTCGTCCCTTTCCAGCTTTTGAGTGAATTGCTGCAACATG-3'.

The Y138L primers were:

5'-CGAACTGGTGTAATGATATGTGCATTATTATTACATCGGGGCAAATTTTTAA-3' and

5'-TTAAAAATTTGCCCCGATGTAATAATAATGCACATATCATTACACCAGTTCG-3'.

The G129E primers were:

5'-ATTCACTGTAAAGCTGGAAAGGAGCGAACTGGTGTAATGATATGTG-3' and 5'-CACATATCATTACACCAGTTCGCTCCTTTCCAGCTTTTGAGTGAAT-3'. Mutations were verified by sequencing. The different PTEN variants were then subcloned into pCS2+ vector for *Xenopus* experiments or baculoviral expression vectors for protein phosphatase activity experiments.

PTEN siRNA resistant version was synthesized by GeneScript, using the following sequence:

ATGACAGCCATCATCAAAGAGATCGTTAGCAGAAACAAAAGGAGATATCAAGAGG
ATGGATTCGACTTAGACTTGACCTATATTTATCCAAACATTATTGCTATGGGATTTCC
TGCAGAAAGACTTGAAGGCGTATACAGGAACAATATTGATGATGTAGTAAGGTTTTT
GGATTCAAAGCATAAAAACCATTACAAGATATACAATCTTTGTGCTGAAAGACATTA
TGACACCGCCAAATTTAATTGCAGAGTTGCACAATATCCTTTTGAAGACCATAACCC
ACCACAGCTAGAACTTATCAAACCCTTTTGCGAGGACTTAGATCAGTGGCTAAGTGA
AGATGACAATCATGTTGCAGCAATTCACTGTAAAGCTGGAAAGGGACGAACTGGTG
TAATGATATGTGCATATTTATTACATCGGGGCAAATTTTTAAAGGCACAAGAGGCCC
TAGATTTCTATGGGGAAGTAAGGACCAGAGACAAAAAGGGAGTAACTATTCCCAGT
CAGCGAAGATACGTCTACTACTACAGCTACCTGTTAAAGAATCATCTGGATTATAGA
CCAGTGGCACTGTTGTTTCACAAGATGATGTTTGAAACTATTCCAATGTTCAGTGGC
GGAACTTGCAATCCTCAGTTTGTGGTCTGCCAGCTAAAGGTGAAGATATATTCCTCC
AATTCAGGACCCACACGACGGGAAGACAAGTTCATGTACTTTGAGTTCCCTCAGCCG
TTACCTGTGTGTGGTGATATCAAAGTAGAGTTCTTCCACAAACAGAACAAGATGCTA
AAAAAGGACAAAATGTTTCACTTTTGGGTAAATACATTCTTCATACCAGGACCAGAG
GAAACCTCAGAAAAAGTAGAAAATGGAAGTCTATGTGATCAAGAAATCGATAGCAT
TTGCAGCATCGAACGAGCGGACAACGACAAGGAATATCTAGTACTTACTTTAACAA

AAAATGATCTTGACAAAGCAAATAAAGACAAAGCCAACCGATACTTTTCTCCAAAT
TTTAAGGTGAAGCTGTACTTCACAAAAACAGTAGAGGAGCCGTCAAATCCAGAGGC
TAGCAGTTCAACTTCTGTAACACCAGATGTTAGTGACAATGAACCTGATCATTATAG
ATATTCTGACACCACTGACTCTGATCCAGAGAATGAACCTTTTGATGAAGACCAACA
CACGCAGATAACGAAAGTCTGA

The changed nucleotides are highlighted in blue and siRNA recognition sites are underlined.

For viral infection of hTERT-RPE1 cells, siRNA-resistant 3Flag-PTEN constructs were generated via Gateway cloning (Life Technologies) using the following primers: 5'-GGGGACAACTTTGTACAAAAAAGTTGCCACCATGGACTACAAAGACCATGACGGTG-3' and 5'-GGGGACAACTTTGTACAAGAAAGTTGGGTATCAGACTTTCGTTATCTGCGTGTG-3' and inserted into pLEX 307 vector (Addgene). The human 3Flag-DVL2 constructs for generating hTERT-RPE1 stable cell lines were also obtained using the QuikChange site-directed mutagenesis kit from Agilent in a pCAGIP vector. The primers used to generate the DVL2 S143A mutant were:
5'- CCACCCTAATGTGTCCAGCGCACATGAGAATCTGGAGCCTG-3' and
5'-CAGGCTCCAGATTCTCATGTGCGCTGGACACATTAGGGTGG-3' (mutated codons underlined).
All constructs used in the study were fully sequenced-verified. Furthermore, the behavior of PTEN mutant constructs cloned into the pLEX 307 vector and used in Supplementary Figure 10d were confirmed by analyzing the levels of AKT phosphorylated on Serine 473 in U-87 MG cells, as previously reported[1].

Lentivirus preparation and infection of hTERT-RPE1 cells

Lentiviral production and infection of hTER-RPE1 cells was performed as previously described[2]. Infectious particles were produced in 293T cells by co-transfection with pLEX 307 3Flag-PTENr vectors, psPAX2 (Addgene) and pMD2.G (Addgene) and pLEX 307-EFGP-NLS (EGFP with nuclear localization signal) as a control using Lipofectamine 2000 (Life Technologies). Viral media, containing DMEM with 4% FBS was collected after 48 h and 72 h and filtered. Infectious particles were determined using LentiXqRT-PCR Titration Kit (Clontech, cat#631235). hTERT-RPE1 cells were plated at a density of 1.75 x 10^5 cells/well in 6-well dishes and transfected the next day with Control or PTEN siRNAs. Twenty-four hours post-transfection with RNAi, cells were infected with lentiviral media in the presence of 5 µg/mL hexadimethrine bromide. After 24 h, infected cells were starved in DMEM (with

Penicillin/Streptomycin) without serum for another 48 h and then lysed in TNTE buffer (50 mM Tris-HCl, 150 mM NaCl, 1 mM EDTA, 0.5% Triton X-100), supplemented with protease inhibitors and processed for immunoblotting.

Flow Cytometry analysis

For Flow Cytometry (FACS) analysis, hTERT-RPE1 cells transfected with siCTL or siPTEN were plated in 6-well dishes, and starved for ~40 h before stimulation with serum to induce cilia disassembly. At different time points post-serum addition cells were trypsinized and resuspended in PBS before fixation with Paraformaldehyde at 4%. Cells were permeabilized with methanol before staining with Propidium iodide at 40 μg/ml and treated with Ribonuclease A (100 μg/ml) for 30 min at 37 degrees. Samples were sorted and data collected using a Gallios (Beckman Coulter) Flow Cytometer. Data was analyzed and graphs generated using the Kaluza Flow Analysis software (Beckman Coulter). As control to detect cell cycle entry and inhibition, untransfected cells were treated with DMSO and Alisertib (5 μM) 2 h prior serum stimulation. DMSO and Alisertib were replenished at the time of serum addition and again after 7 h of serum. These samples were processed for FACS after 24 h of serum.

Purification of bacterially expressed PTEN protein

Wild-type (WT) PTEN containing residues 7-353 and DACS (D92A/C124S) versions, were amplified by PCR and cloned into modified pET30M polyHis-GST expression vector via NcoI and NotI restriction sites. Both PTEN versions were overexpressed in E. coli BL21 (DE3) as a poly histidine-GST tagged fusion. Expression was induced with 1 mM IPTG overnight at 17°C. Protein was purified using Ni NTA chelating chromatography. Fractions containing His-GST-PTEN protein were pooled. Each of these proteins was further purified using glutathione sepharose 4B. All site-directed mutagenesis carried out in PTEN were performed by conventional PCR techniques. Mutations were confirmed by DNA sequencing.

***In vitro* Protein interaction Assay**

In vitro protein interaction experiments were carried out with bacterially expressed GST or His-GSTPTEN WT or DACS version with approximately equimolar amount of GST or GSTPTEN incubated with 0.3 μg cell lysates of hTERT-RPE1 cells either transfected with pCMV5 vector or

3Flag-DVL2 WT or S143A mutant, the cell lysates were diluted in 1 ml TNTE containing 0.1% Triton-X-100, 50 mM Tris HCl pH 7.4, 150 mM NaCl, 1 mM EDTA and incubated at 4°C on a shaker for 1 hour. After washing GST or GST-PTEN beads five times, 50 µl sample buffer was added and boiled, samples were resolved on SDS-PAGE and immunoblotted with either M2 anti-Flag and anti-His/GST antibodies.

PTEN purification from baculovirus system

Spodoptera frugiperda (Sf9) cells were used as an expression system for PTEN or its corresponding mutant versions. An N-terminal 6xHis tag followed by a Tobacco Etch Virus (TEV) cleavage site was used to isolate PTEN after a 48 h infection at 27 ^{o}C. Cell pellets were produced after washing in PBS and centrifugation. Cells were lysed via sonication for 5 m in Buffer A: 20 mM Tris pH 8.0, 300 mM NaCl, 10 mM imidazole pH 8.0, 5% glycerol, 2 mM β-mercaptoethanol, 0.5% Triton X-100, with one complete-EDTA free protease inhibitor tablet added (Roche) per 50 ml of buffer. This lysate was subsequently centrifuged for 45 min at 140,000 x *g*. Supernatant was passed through a 0.45 µm filter (Sartorius Biotech) and loaded onto a 5 ml HiTrap FF column (GE Healthcare). The column was washed with 30 mM imidazole before being eluted with a 0-100% gradient of Buffer B (20 mM Tris pH 8.0, 100 mM NaCl, 5% glycerol, 200 mM imidazole, 2 mM β-mercaptoethanol). His-tagged TEV protease was added to PTEN containing fractions, and this solution was dialysed in 4 L of Buffer C (20 mM Tris pH 8.0, 200 mM NaCl, 5% glycerol, 2 mM TCEP) at 4°C for 12 hours using a 10,000 MWCO Snakeskin Dialysis membrane. A 1:20 w/w ratio of PTEN:TEV was used for cleavage. By passing the dialysed solution over a 5 ml HiTrap FF column TEV was removed. FloWThrough from the HiTrap column was then diluted 1:1 with Buffer D (20 mM Tris pH 8.0, 10% glycerol, 1 mM DTT), and loaded onto a 5 ml HiTrap Q Column (GE Healthcare), pre-equilibrated in Buffer E (20 mM Tris pH 8.0, 50 mM NaCl, 10% glycerol, 1 mM DTT). Protein was then eluted with a gradient of 0-100% of Buffer F (20 mM Tris pH 8.0, 1 M NaCl, 10% glycerol, 1 mM DTT). Pooled fractions were concentrated with an Amicon 10,000 MWCO centrifugal filter (Millipore) and then injected onto a Superdex 75 16/60 gel filtration column, pre-equilibrated in Buffer G (20 mM HEPES pH 7.4, 200 mM NaCl, 2 mM TCEP). Fractions were collected, concentrated to at least 1.5 mg/ml using an Amicon 10,000 MWCO centrifugal filter (Millipore), aliquoted, and frozen in liquid nitrogen and stored at -80°C prior to experimentation.

Protein Dephosphorylation

Due to the low activity of phosphorylated native PTEN, the purified PTEN protein was incubated at 30°C for 90 minutes with Lambda Protein Phosphatase (NEB P0753) as per the manufacturer's instructions (approximately 50,000 units per reaction). The reaction solution (1500 µL) was then diluted to 20 ml of Buffer D and purified using a 5 ml HiTrap Q Column as described above. PTEN containing fractions were then concentrated to a 2 ml volume using an Amicon 10,000 MWCO centrifugal filter (Millipore) and then injected onto a Superdex 75 10/300 equilibrated and run in Buffer G. Protein containing fractions were concentrated and frozen in liquid nitrogen for subsequent analysis. Phosphorylation status was checked with intact mass spectrometry.

Equipment and Settings

The images were acquired using Leica DMIRE2 microscope, equipped with Hamamatsu EM-CCD C9100-13 digital camera and following objectives: PL FLUOTAR 20x/0.5, PL APO 63X/1.32-0.6, oil, HCX PL APO 40x/.1.25-0.75 oil. The pixel dimensions were the following: for 20x objective x and y were 0.4 µm per px, z was 1 µ per px; for 40x objective x and y were 0.2-0.25 µm per px, z was 0.5 µm per px; for 63x objective x and y were 0.13 µm per px, z was 0.5 µm per px. The image resolution was 512x512 px with original image bit depth of 16 bits. The image processing, time-lapse settings and fluorochromes are described in the correspondent **Methods** sections.

Immunoblotting images were acquired using ChemiDoc^{TM} MP Imaging System (BioRAD) and Image Lab^{TM} Software (BioRAD).

Supplementary References

1. Tibarewal, P. *et al.* PTEN protein phosphatase activity correlates with control of gene expression and invasion, a tumor-suppressing phenotype, but not with AKT activity. *Science signaling* **5**, ra18, doi:10.1126/scisignal.2002138 (2012).
2. Hirsch, C. L. *et al.* Myc and SAGA rewire an alternative splicing network during early somatic cell reprogramming. *Genes & development* **29**, 803-816, doi:10.1101/gad.255109.114 (2015).
